# Public anxiety through various stages of COVID-19 coping: Evidence from China

**DOI:** 10.1371/journal.pone.0270229

**Published:** 2022-06-16

**Authors:** Yangyang Wu, Ting Zhang, Ziwen Ye, Kai Chen, J. van der Kuijp, Xue Sun, Guoyi Han, Yi Zhao, Yang Liu, Lei Huang

**Affiliations:** 1 School of the Environment, Nanjing University, Xianlin Campus, Nanjing, China; 2 Department of Environmental Health Sciences, Yale School of Public Health, New Haven, CT, United States of America; 3 Yale Center on Climate Change and Health, Yale School of Public Health, New Haven, CT, United States of America; 4 Department of Environmental Science and Public Policy, Harvard University, Cambridge, MA, United States of America; 5 Stockholm Environment Institute, Stockholm, Sweden; 6 Gangarosa Department of Environmental Health Rollins School of Public Health, Emory University, Atlanta, GA, United States of America; Universidade Federal do Rio Grande do Sul, BRAZIL

## Abstract

As countries underwent the initiation, peak, post-peak, and early vaccination stages of COVID-19, the changing risk perception, coping behaviors and corresponding psychological stress experienced by the public over time was rarely reported. We conducted a national scale panel study using social-psychological data collected from 5,983 questionnaires to investigate the interactions between anxiety level, risk perception and coping behavior during different stages of COVID-19 in China. We found that sustained perceiving worries of being infected, first due to domestic and then global pandemic, contributed to the persistent high proportion of respondents with anxiety disorders which even gradually increased over time (56.1% during initiation to 60.4% during early vaccination). Gender was the strongest predictor of anxiety at all stages, with females having less confidence in COVID-19 control and always suffering from much higher anxiety levels than males even during the post peak stage. Excessive protective behavior and frequency of access to COVID-related news also contributed to public anxiety. Additionally, public risk perception was significantly associated with their willingness to vaccinate. The findings verify the feasibility of taking stage-specific and gender-based risk communication strategies to alleviate the pandemic-related public anxiety and promote vaccination by influencing public risk perception and guiding coping behaviors.

## Introduction

Numerous studies have shown that COVID-19 not only poses a fatal physical health threat but also triggers a global mental health crisis [1]. Mental disorders reduce productivity and quality of life, and can lead to serious health consequences, such as suicide [2–4]. In some countries such as Japan, suicides related to mental disorders (such as anxiety disorders) even exceeded deaths directly caused by COVID-19 [5]. A global retrospective study evaluated the psychological impact of the implementation of quarantine due to different pandemics/epidemics (i.e., SARS, Ebola, MERS, etc.), and indicated that strict prevention/control measures (longer isolation periods) and negative risk perceptions (such as excessive worries about being infected) had important psychological effects [6]. During the first lockdown in UK, those who resisted or felt suffering from the lockdown experienced a significant increase (over 50%) in anxiety and depression during the pandemic, and alcohol consumption in this segment increased by over 20% [7]. Personal coping behaviors such as excessive frequency of access to COVID-related news could lead to acute stress, which were also positively correlated with personal risk perception [8, 9].

Till now, most studies concerning the personal risk perception levels and mental health issues during COVID-19 have been conducted within the same stage of the pandemic. Thus, they were unable to capture the changes of public risk perception level and corresponding psychological stress among different stages of the pandemic in a given area [9–11]. For example, three surveys in a national study in the US were all conducted in the peak stage of the pandemic [9]. Also, our study considered the contribution of individual coping behaviors. Previous studies had limitations in that some research only explored the relationship between risk perception or coping behavior and anxiety, ignoring the inner connections among them [8, 12, 13], while others only focused on the changes in anxiety level and related socio-demographic characteristics [14–16]. Finally, public risk perception level of the COVID-19 will further affect whether they are willing to be vaccinated. The fact that a growing number of people are receiving vaccination also necessitates more understanding about the impact of vaccination on anxiety.

COVID-19 first broke out in China but has been largely under control except for some small waves of outbreaks since April, 2020 [17, 18], when the 76-day lockdown of Wuhan, the original epicenter of the COVID-19 crisis, finally ended. In this study, we carried four social-psychological questionnaire surveys between February 2020 and January 2021 and used the generalized linear mixed model (GLMM) to explore the contribution of risk perception and coping behavior to anxiety. Our study period covered the typical initial, peak and post-peak stages of the pandemic and the first vaccination stage of the COVID-19 crisis across China. To the best of our knowledge, this is the first nationwide panel study investigating the changes of public risk perception levels about the COVID-19 and corresponding coping behaviors over time as well as detecting their changing contributions to anxiety levels in each stage.

## Materials and methods

### Study design and participants

**Fig 1:**
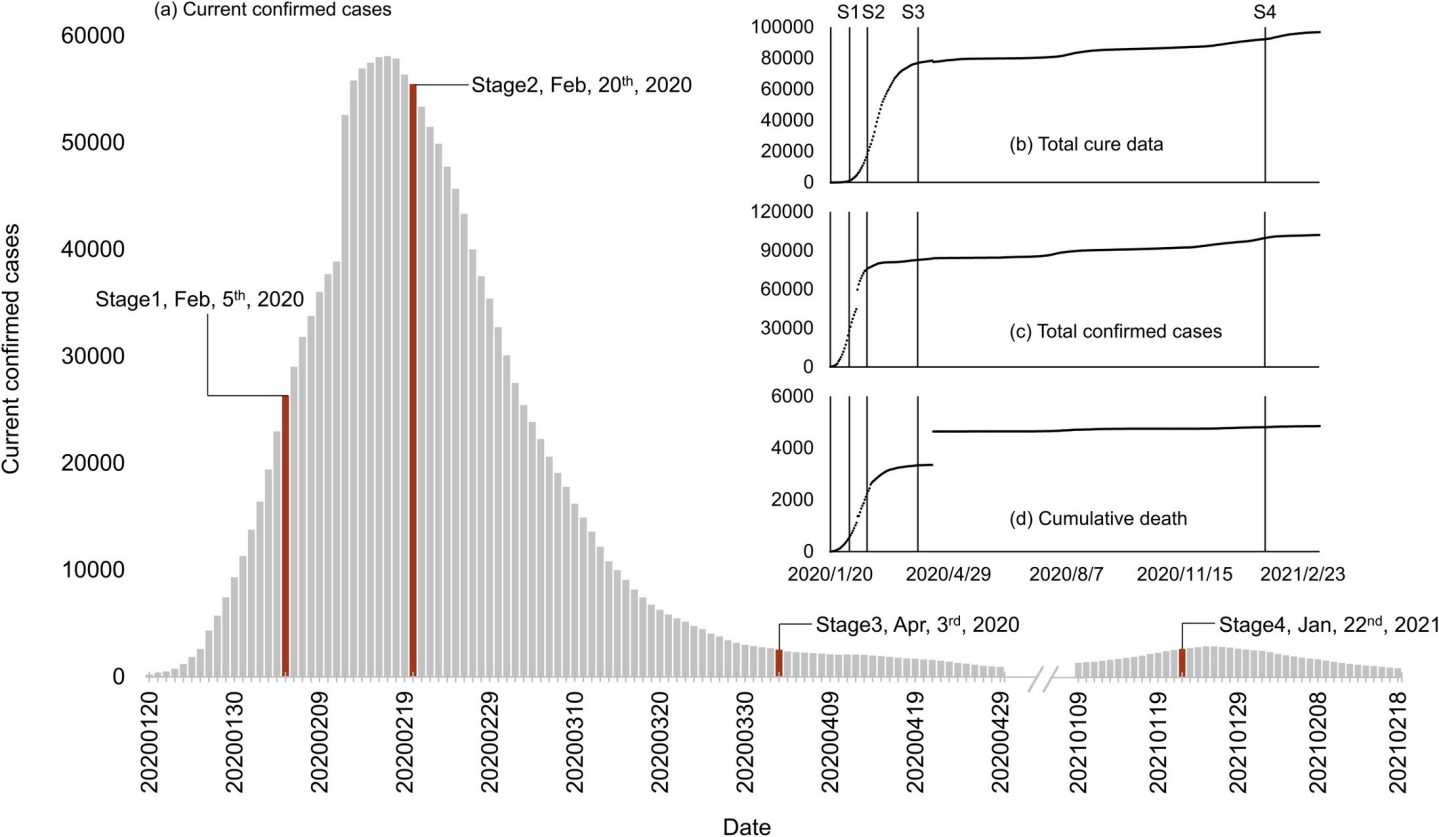
COVID-19 statistics in mainland China during the study period (a) Current confirmed cases, (b) total cure data, total confirmed cases and (d) cumulative deaths. Data source: http://www.nhc.gov.cn/xcs/yqtb/list_gzbd.shtml.

Fig 1 demarks the four stages. The first three surveys were conducted separately in early February (February 5^th^ -February 10^th^), late February (February 20^th^ -February 25^th^) and early April 2020 (April 3^rd^ -April 8^th^), corresponding to the initial (*Stage 1*), peak (*Stage 2*), and post-peak (*Stage 3*) of the outbreak in China. We carried out the fourth survey in late January 2021 (January 22nd—January 26^th^). By the initial stage (Stage 1), the human-to-human transmission has been verified and reported before long, the daily confirmed cases quickly increased (Fig 1) but the new cases were mainly detected in Wuhan and cities around it. In the peak phase (Stage 2), the virus spreads massively outside Wuhan, and the total number of confirmed cases peaks. In the post-peak stages (Stage 3 and 4), the daily confirmed cases nationwide had largely dropped, signifying the decreasing pandemic activity. However, there were still additional small pandemic waves. Specifically, the *Stage 4* was also a post-peak phase with small-scale outbreaks in the north and northeast of China and the beginning of mass vaccination at the same time.

The surveys were taken via a free online questionnaire applet (Wenjuanxing, https://www.wjx.cn/). We got the informed consent of the respondents at the beginning of each questionnaire and promised that they could freely decide whether to continue the survey or quit whenever they want to. Thus, dropping in the number of the respondents throughout this study was completely random and introduced no selection bias. Each respondent would leave the last four digits of their phone number or a code name created by themselves in the questionnaire to ensure continuous tracking in the questionnaire. We compared the risk perceptions, coping behaviors and anxiety levels of the interviewees in the last three stages with those in the first stage respectively to detect population-level changes in above factors over stages. The Ethics Committee of School of the Environment, Nanjing University approved the protocol of this study. We confirmed that all methods were carried out in accordance with relevant guidelines and regulations as well as performed in accordance with the Declaration of Helsinki. Informed verbal consent was obtained from participants or from a parent and/or legal guardian via telephone inquiries if the participant was under 18.

### Measures

We investigated the changes in respondents’ risk perception levels of the COVID-19 in each stage (including attention, worries, belief in controllability and the influence of the COVID-19, etc.), and collected the statistics about the degree of coping behaviors taken by each respondent, including protective behavior (wearing mask, washing hand, etc.), outdoor activity (for shopping, for work, etc.), and frequency of access to COVID-related news (Table 1). The score of protective behavior was the total number of behaviors the respondent carried out. The score of each outdoor activity and frequency of access to COVID-related news were determined according to the frequency of these activities (scoring 1~5, and the higher the score, the more frequent they conducted the activity). The final level of outdoor activity was the sum of frequency of each activity. The enrolled risk perception about COVID-19 were also listed in Table 1. A five-point Likert scale (scoring: 1~5, and the higher the score, the higher the agreement) was used to investigate the respondent’s risk perception. We used the score of a question or the sum of scores of multiple questions that was/were used to assess a given perception to represent the level of this risk perception. Crown-Crisp index phobic anxiety scale (score: 0~16) was used to distinguish between people with high anxiety or phobias and healthy participants [19]. The Crown-Crisp phobia index measures common symptoms of phobic anxiety. With the validation in psychiatric outpatient clinic settings, this index has been widely applied in estimating the public’s phobic anxiety [19–21]. When reaching a score of six or more, the respondent would be defined as bearing high anxiety level [19–21]. Socio-demographic variables included gender, age, education, occupation, and location. In *Stage 3* questions about the cognition of the controllability and the attention paid to the foreign pandemic were enrolled. In *Stage 4*, we further added questions of their vaccination status and attitudes towards domestic vaccines.

**Table 1:** Risk perceptions and coping behavior variables investigated at various stages.

Variables	Questions	Stages
**Risk perceptions**		
Domestic attention	How much do you pay attention to COVID-19 in the country?	All
Global attention	How much do you pay attention to global pandemic?	Stage 3, 4
Trust	How much do you believe that infected patients can get adequate medical resources?	Stage 1, 2, 3
Domestic controllability	How much do you think that the outbreak of COVID-19 can be effectively controlled in China?	All
Global controllability	How much do you think that the outbreak of COVID-19 can be effectively controlled globally?	Stage 3, 4
Understanding	Virus Source	Stage 1, 2, 3
	Transmission channels and mechanisms	Stage 1, 2, 3
	Treatment method	Stage 1, 2, 3
	Infection symptoms	Stage 1, 2, 3
Interference	How much do you think COVID-19 interferes with your normal life?	All
Vaccine trust	How much do you trust vaccine against COVID-19 that produced in China?	Stage 4
Worried	Domestic pandemic: Worry about being infected	All
	Global pandemic: Cold chain food	Stage 4
	Global pandemic: Imported goods	Stage 4
	Global pandemic: Study abroad	Stage 4
	Global pandemic: Incomes	Stage 4
	Global pandemic: Reunite	Stage 4
**Coping behavior**		
Access to information	How often do you check news about COVID-19 every day	All
Outdoor activity	How often do you go out each week	All
	1, Outdoor-work	
	2, Outdoor-dinner	
	3, Outdoor-visit	
	4, Outdoor-shopping	
	5, Outdoor-others	
Protective behavior	Do you take the following protective measures against COVID-19	All
	1, Behavior-Hoard masks or goggles	
	2, Behavior-Hoard medicines	
	3, Behavior-Open windows for ventilation	
	4, Behavior-Indoor disinfection	
	5, Behavior-Wash hands frequently	
	6, Behavior-Reduce going out	
	7, Behavior-Avoid people with colds and coughs	
	8, Behavior-Avoid gathering activities	
	9, Behavior-Avoid contact with people in high-risk areas	
	10, Behavior-Exercise	
	11, Behavior-Other	
	12, Behavior-None	
Community closed	Has the village / community where you lived daily been closed and outsiders are prohibited?	Stage 1, 2, 3
Precaution extent	The prevention degree you think that have taken to fight against COVID-19?	Stage 1, 2, 3

Notes: ***All*** means the questions were asked in each stage. Controllability means belief in the controllability. Access to information means the frequency of access to COVID-related news.

### Statistics analysis

We conducted four rounds of questionnaire surveys and explored the relationship of the public’s risk perception, behavioral change, and anxiety regarding the COVID-19 via generalized linear mixed model (GLMM). In the basic model, we controlled for the number of people moved to Wuhan to where the respondent was then by on Jan. 20^th^, 2020 (the day when the human-to-human transmission was firstly announced, normalized), daily detected cases (normalized), and variables related to the socio-demographic characteristics including gender, age, education, community status, area and occupation. Firstly, we investigated the spatiotemporal changes of the public anxiety, risk perception and coping behaviors. Secondly, we explored the associations between these factors and the socio-demographic characteristics of the participants, respectively. Thirdly, we respectively detected the contributions of risk perceptions and coping behaviors to anxiety levels. In addition, we quantitatively evaluate the impact of domestic and global situation of the pandemic on public anxiety over time. Based on the results of descriptive statistics and GLMM, we found that public risk perception, coping behavior and anxiety level showed significant gender differences. In order to test the robustness of the model results and explore the contribution of males and females’ risk perception and coping behavior to anxiety levels, we conducted a subgroup analysis by gender (S1 Table). Please see S1 Text for more details about GLMM. Additionally, we performed One-way analysis of variance (ANOVA) to explore whether the differences between the vaccinated and unvaccinated groups were significant or not. We also use Welch’s test and Brown-Forsythe test to verify whether the changes in risk perception and coping behavior between stages were significant. Cronbach’s alpha, Kaiser-Meyer-Olkin (KMO) and Bartlett’s sphericity tests were carried out to test the reliability and validity of the questionnaires (S2 Table). Statistical significance was defined as a *p*-value less than 0.05. Statistical analysis was conducted in R version 4.0.0 (R Foundation for Statistical Computing, Vienna, Austria).

## Results

### Descriptive statistics

A total of 5,983 valid questionnaires (effective sampling: 87.3%) were used for analysis. Male and female respondents accounted for 43.0% and 57.0%, respectively. 26.9% of respondents didn’t have a bachelor’s degree and 45.4% of the sample is comprised of students. Respondents aged 18–25 accounted for 51.6%. More detailed demographic information is shown in S3 Table and S2 Text.

### Spatiotemporal changes of risk perceptions, coping behaviors and anxiety

**Fig 2:**
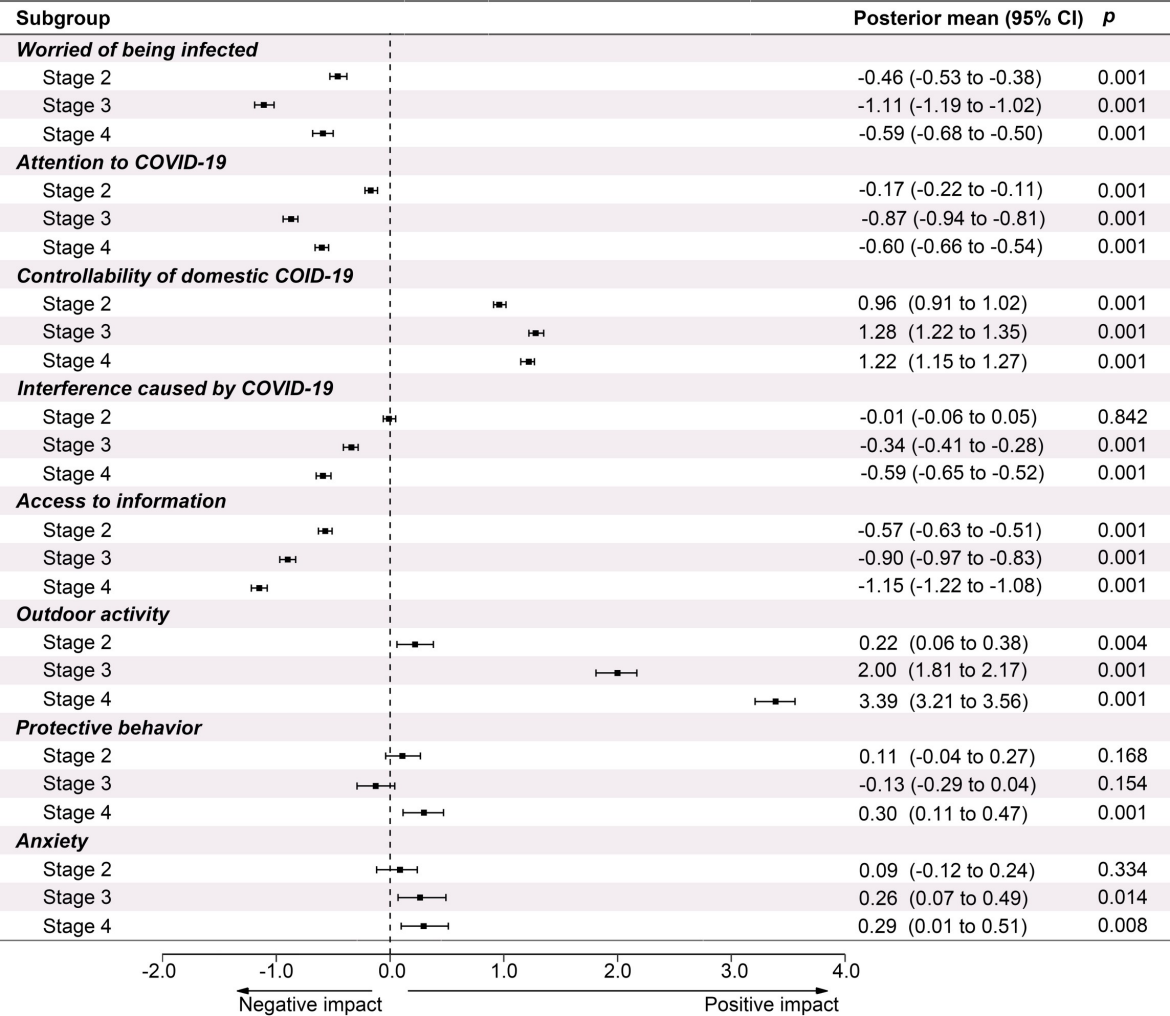
Changes in respondents’ risk perceptions, coping behaviors and anxiety level (95% CI) in different stages compared with Stage 1. Controllability means belief in the controllability of domestic COVID-19 crisis. Access to information means the frequency of access to COVID-related news.

From *Stage 1* to *Stage 3*, the respondents’ attention to the domestic outbreak continued to decline significantly but their belief in the controllability of domestic COVID-19 kept increasing (Fig 2 and S4 Table and S1 Fig). In *Stage 4*, with a new wave of outbreak in part of China, the attention increased slightly (Post. mean -0.60, 95% CI -0.66 to -0.54, *p* < 0.001) while the level of the belief in controllability maintained at a high level (Post. Mean 1.22, 95% CI 1.15 to 1.27, *p* < 0.001). In *Stage 4*, up to 34.1% and 35.4% of respondents expressed their worries about the imported cold chain foods and goods, which may carry coronavirus. The proportion of respondents believing that the global pandemic was completely controllable was less than 5% (S2 Fig and S5 Table). The frequency people searching for COVID-19-related information continued to decrease. As time passed, the frequency of outdoor activities increased significantly while increase in the degree of *protective behavior* was significant only in *Stage 4* (Post. mean 0.30, 95% CI 0.11 to 0.47, *p* < 0.001) when compared with *Stage 1* (S4 Table and Figs S1 and 2). Respondents with high anxiety accounting for 56.1%, 57.6%, 60.4% and 60.0% in the four stages, respectively (Table 1). Significant increases were detected from *Stage 1* to *3* (Post. mean 0.26, 95% CI 0.07 to 0.49, *p* < 0.05), and from *Stage 1* to *4* (Post. mean 0.30, 95% CI 0.10 to 0.51, *p* < 0.01) (S6 Table). The percentage of females with anxiety symptoms and its average anxiety score at each stage were much higher than those of males (Table 2).

**Table 2:** Anxiety level statistics considering gender differences in each stage.

Stage	*Stage1*		*Stage2*		*Stage3*		*Stage4*	
***All respondents***								
respondents with high anxiety (%)	56.2%		57.6%		60.4%		60.0%	
Mean score [SD]	6.03 [2.84]		6.13 [3.01]		6.30 [3.02]		6.33 [2.97]	
***Gender***	Male	Female	Male	Female	Male	Female	Male	Female
≥6 (%)	48.3%	62.4%	50.4%	63.0%	54.3%	64.5%	51.0%	67.0%
Mean score [SD]	5.54 [2.79]	6.43 [2.81]	5.62 [3.11]	6.51 [2.88]	5.85 [3.03]	6.61 [2.98]	5.73 [2.98]	6.79 [2.89]

The mean anxiety score of all respondents and the percentage of respondents with high anxiety (≥6); mean anxiety score of high anxiety respondents (≥6); the percentage of male or female respondents with high anxiety (≥6) and their mean anxiety score.

The average level of coping behavior was relatively weaker in Northeast China and stronger in Southwest China which also had higher risk perceptions overall (S7 Table). Respondents in Northwest China displayed statistically higher anxiety level than the rest regions (p < 0.001) from *Stage 1* to *Stage 3* (S8 Table). No significant spatial difference was detected in anxiety levels in *Stage 4* even though the average anxiety score of respondents in Northeast reached 7.44 during this stage.

### Difference between socio-demographic sub-groups in risk perceptions, coping behaviors and anxiety

**Fig 3:**
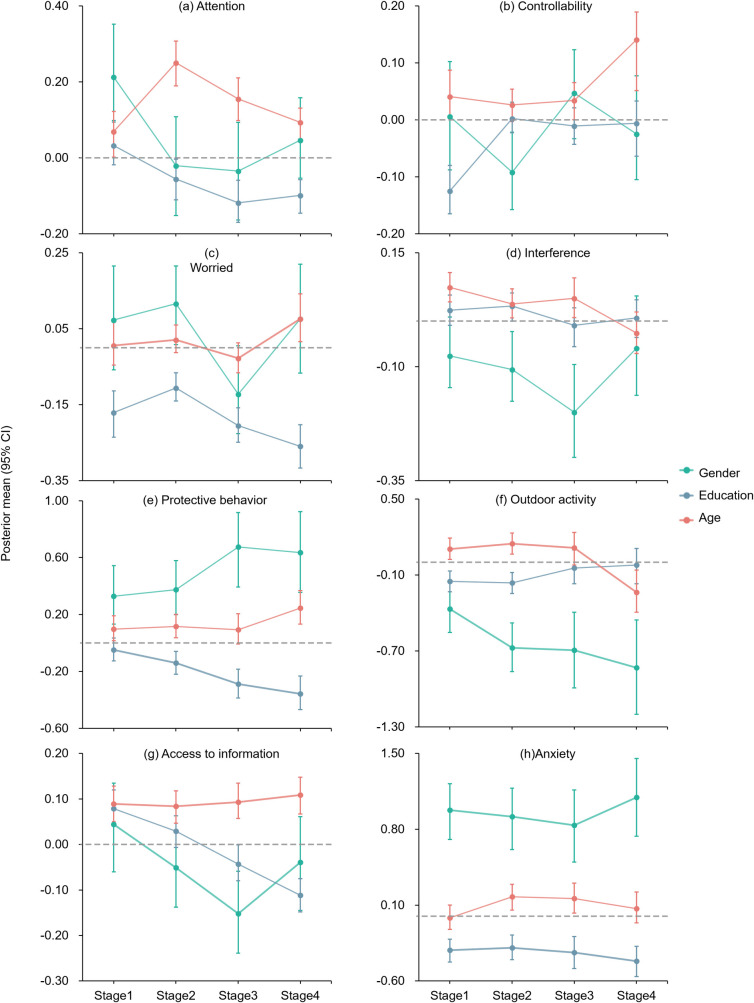
The impacts of individual characteristics on respondents’ risk perceptions (a, b, c, d), coping behaviors (e, f, g) and anxiety level (h) in different stages. The dot represents the Post. means of female vs. male, the senior vs. the junior, and the more educated vs. the less educated and the bars represent the upper and lower levels of 95% confidence intervals of the Post. means. Controllability means belief in the controllability of domestic COVID-19 crisis. Access to information means the frequency of access to COVID-related news.

We further examined the socio-demographic characteristics of respondents’ risk perceptions at each stage (Fig 3). The elderly always paid more attention to COVID-19 related information especially during the peak stage (Post. mean 0.25, 95% CI 0.19 to 0.31, p < 0.001) while the less educated people were more worried about being infected (p < 0.001). The less educated also paid more attention to the new waves of outbreak (Post. mean -0. 10, 95% CI -0.15 to -0.06, p < 0.001). The female was also more worried about being infected (Post. mean 0.12, 95% CI 0.01 to 0.21, p < 0.05) and had less confidence in domestic control of COVID-19 (Post. mean -0.09, 95% CI -0.16 to -0.02, p < 0.01) than the male in the peak stage. Further analysis showed that the less educated people and females tended to implement more comprehensive protective behaviors and reduce outdoor activities. Gender difference was the only factor placing significant impact on the above mentioned two behaviors at each stage.

Most importantly, the less-educated and female respondents were more likely to suffer from anxiety disorders (S9 Table). In the first three integrated stages, the elderly was more inclined to have higher anxiety levels. The contribution of gender difference on anxiety far exceeded that of risk perceptions, coping behaviors and other individual factors (Post. mean 0.84–1.10, *p* < 0.001). The average anxiety level of females was much higher than that of males in each stage of study (Table 2). Education level was another factor that interfered with anxiety throughout the process, although its influence was much lower than the impact from gender difference. The influence of gender difference (Post. mean 1.10, 95% CI 0.74 to 1.45, *p* < 0.001) and education levels (Post. mean -0.42, 95% CI -0.56 to -0.28, *p* < 0.001) on anxiety both peaked in *Stage 4*.

### Contributions of risk perception and coping behaviors to anxiety

Worry about being infected was always the strongest predictor for anxiety levels among risk perceptions in the first three integrated stages (Post. mean 0.56, 95% CI 0.49 to 0.63, *p* < 0.05) (S9 Table). In *Stage 4*, respondents’ worries about global pandemic was significantly related to higher level of anxiety (Post. mean 0.54, 95% CI 0.31 to 0.78, *p* < 0.001), and even exceeded the influence related to worry of being infected by the domestic outbreak. Additionally, people’s *attention* to the pandemic (Post. mean 0.13, 95% CI 0.02 to 0.25, *p* < 0.05) and the degree to which it *interfered* with their daily life (Post. mean 0.11, 95% CI 0.01 to 0.21, *p* < 0.05) were also related to their anxiety levels throughout the first three integrated stages.

**Fig 4:**
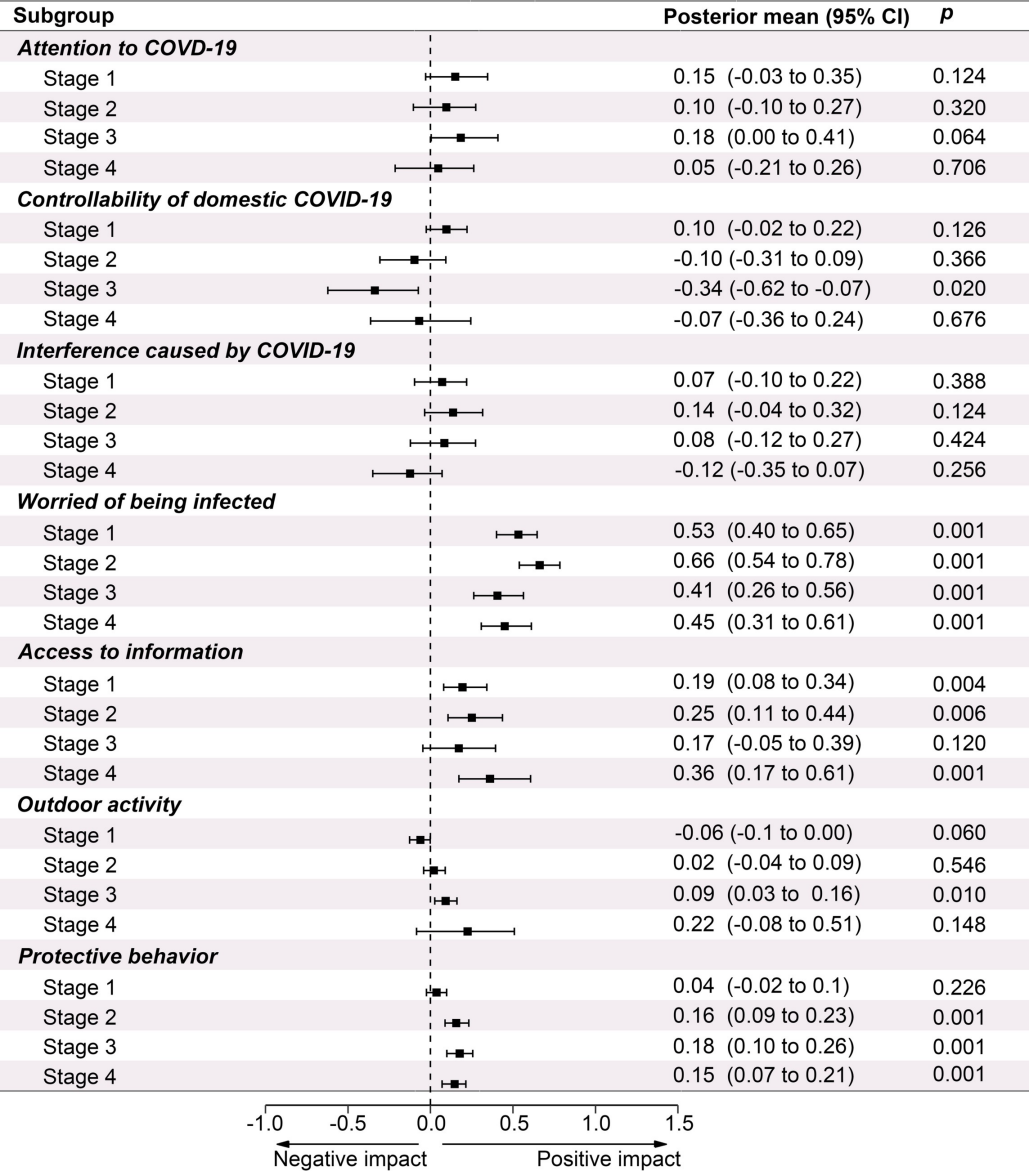
The impacts of respondents’ risk perceptions and coping behaviors on anxiety levels on different stages of the COVID-19 in mainland China. Controllability means belief in the controllability of domestic COVID-19 crisis. Access to information means the frequency of access to COVID-related news.

Excessive *protective behaviors* (Post. mean 0.11, 95% CI 0.07 to 0.15, *p* < 0.001) and frequent *access to COVID-19 related information* were associated with increased anxiety (Post. mean 0.18, 95% CI 0.09 to 0.26, *p* < 0.001) at nearly each stage (Fig 3 and S9 Table). As the frequency of access to COVID-related news considerably decreased in *Stage 3*, its association with anxiety was no longer significant as it was in other *stages* with the most notable association appeared *in Stage 4* (Post. mean 0.36, 95% CI 0.17 to 0.61, *p* < 0.001). Frequency of *outdoor activities* notably increased in *Stage 3* and was associated with significantly higher anxiety (Post. mean 0.09, 95% CI 0.03 to 0.16, *p* < 0.01) (Figs 2 and 4).

### Vaccine influence

**Fig 5:**
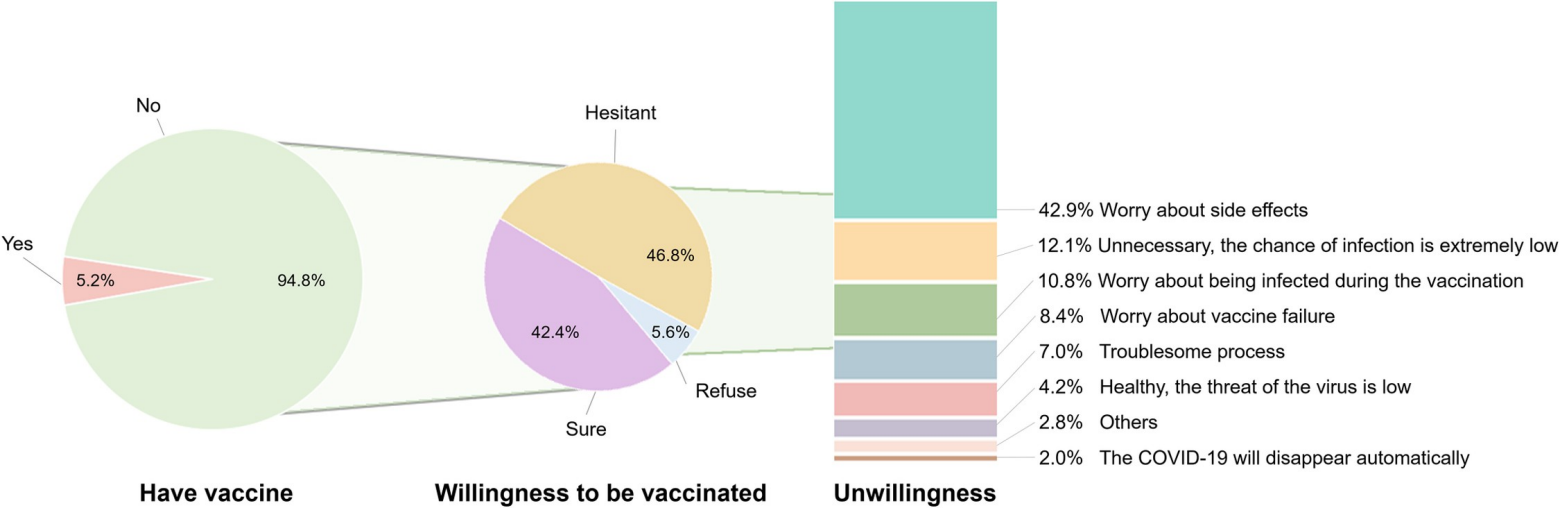
The proportion of respondents who were vaccinated in Stage 4 and the willingness of those who were not vaccinated to vaccinate as well as the reasons for reluctance to vaccinate.

In *Stage 4*, 5.2% of the respondents have had the COVID-19 vaccine (Fig 5). The mean [SD] anxiety score of vaccinated respondents was 6.22 [3.49], lower than that of non-vaccinated respondents (6.34 [2.94]). But the difference was not significant (S10 Table). To unvaccinated respondents, those who were more worried about (F = 5.69, *p* < 0.05), paid more attention to (F = 6.94, *p* < 0.01) the global pandemic and felt more interference from the pandemic (F = 4.76, *p* < 0.05) were more inclined to get vaccinated. 71% of the respondents highly trusted domestic vaccines. For those holding less trust, up to 42.9% of them worried about the side effects of vaccines (S11 Table). Females and less educated people had significantly lower trust in vaccines (S12 Table).

## Discussion

We detected public psychological stress response, corresponding risk perception and coping behavior changing process under special public health events with a focus on quantifying the impact of domestic and global pandemics on public anxiety over time. We concluded that high anxiety caused by COVID-19 has become a persistent and common psychological problem, necessitating stage-specific balanced measures and gender-based risk communication strategies to alleviate. Our research is timely and necessary to remind people to pay attention to mental health throughout the pandemic based on the following key findings.

First, both the respondents’ average anxiety levels and the percentage of respondents with determined high anxiety remained high throughout the four stages. Studies from other countries also reported high levels of anxiety of the public in different periods of the COVID-19 pandemic [7, 14, 22, 23]. Mental illness, including anxiety, is among the main causes of suicide in China [24]. Although the overall suicide rate was relatively low (9.7 per 100,000 people) in China, the total number of suicides each year accounts for approximately 25% of that worldwide [25, 26]. We speculate that the growing anxiety associated with COVID-19 was associated with an increased suicide rate for Chinese. This possibility could be partially verified by the surge in COVID-19-related suicide cases in Japan [5]. In our study, the overall anxiety levels even slightly increased in *Stage 3* and *Stage 4*, or the post-peak stage of the pandemic. It is worth noting that even though many countries are currently in the post-peak stages embracing the decreasing COVID-19-related threat on physical health, the related mental health problems could hardly be overlooked for now.

Second, we noticed that females suffered from notably higher levels of anxiety. Females tend to take more caring responsibilities in and out home, and face more domestic violence and higher unemployment risk during the pandemic, thus they were more likely to be sensitive to external pressures [27, 28]. The high sensitivity of females may drive them to make more negative assessments, suffer more from negative risk perceptions and take more corresponding coping strategies. This could be verified by the fact that females had significantly less frequent outdoor activities throughout the study period and were adopting increasing levels of coping behaviors as the pandemic continued (S3 Text and S13 Table). Together, due to their negative perceptions and excessive coping behaviors, females have much higher levels of anxiety than males. Support for equal employment and income equality, punishment of domestic violence [27], and guidance in adopting appropriate strategies against the pandemic, are warranted to alleviate the high anxiety of females. Regarding other individual factors, the less educated worried about being infected and the elderly people were prone to have higher frequency of access to COVID-related news. In particular, the less educated people easily suffer from mental health crises. Studies in many countries also indicated that a much higher proportion of people with lower education levels or the elderly had high anxiety during the pandemic because they often faced more severe health threats, insufficient financial capacity, etc. [29–31]

Third, multiple risk perceptions were related to anxiety level with worry about being infected being the robust predictor. Sustained worries over the spread of the virus, first domestically and then globally, underlie the persistent high level of anxiety among the public. Since our second investigation, the epicenter of COVID-19 has transferred from China to the US, and then to Europe. Respondents began to pay more attention to foreign pandemic, and thought the controllability of outbreaks in other countries was much lower than that in China. In *stage 4*, the contribution of worries about global pandemic to increased anxiety was even higher than worry about being infected. The negative attitudes towards pandemic status outside of China, together with the present decline in the economy due to COVID-19 and the resultant increase in unemployment [32], the potential infection from imported food or goods [33] and reduced work/study opportunities abroad [34] may jointly lead to increased anxiety for Chinese. It is then reasonable to conclude that, to effectively reduce anxiety of the public, we need to not only overcome the outbreak and spread of the domestic pandemic but also emphasize global cooperation. Measures including medical assistance between countries and the global vaccine sharing are encouraged to bring the pandemic under control as soon as possible. As COVID-19 would last at least in the near future and probably become a chronic seasonal disease in the long run [35], it is necessary to adopt appropriate prevention and control measures as well as public education and publicity measures to reduce the people’s worry of being infected.

Moreover, a number of coping behaviors were able to account for increased public anxiety. Excessive frequency of access to COVID-related news in certain stages contributed to high anxiety disorder. A national study conducted in the US proved that frequent exposure to COVID-19-related media could negatively affect people’s mental health [9]. A previous study confirmed that there were more unverified rumors and exaggerated information on social media, the main source of COVID-19-related information for respondents [36]. Taken together, to diminish anxiety from such sources, the government should take up the responsibility of dispelling rumors and publishing accurate and timely pandemic information. Exaggerated amounts of self-protection measures in the later stages also led to anxiety disorders, especially when the pandemic was basically under control. Under such circumstances, the government may also guide the public to adopt appropriate and less stringent self-protection measures to achieve a balance that weighs physical and mental health.

Finally, the vaccinated respondents had relatively lower levels of anxiety than the nonvaccinated respondents but the difference was insignificant. Previous research claimed that the COVID-19 vaccine offers benefits in reducing the spread of the pandemic [37], thus may contribute to anxiety relief in the long run. However, 52.4% of respondents, especially females and the less educated, still hesitated to get vaccinated for now due to worries about the side effects of vaccination etc. Such worries and hesitancy existed for other vaccines before but were attenuated when their safety and effectiveness were verified [38–41]. Meanwhile, it is also necessary to popularize knowledge of vaccination for the public, especially knowledge about its broad benefits, not only for health concern, but also for the socioeconomic development. With the addressed vaccine enthusiasm and built trust, we could finally achieve large-scale vaccination, bring the COVID-19 under control and eliminate the pandemic-related anxiety [37, 42].

## Limitations

First, we were unable to conduct the first questionnaire survey at the very beginning of the pandemic due to time constraints in designing the questionnaire. Our first phase of research was still carried out in the early stage of the outbreak. Second, due to the online format of the questionnaire surveys, the overall age of the respondents was relatively young while education levels were relatively high. Third, we were unable to recruit subjects completely randomly during the pandemic (the questionnaire company stopped serving, and all citizens could not move freely), so the sampling method has certain limitations, combing the ideas of convenience sampling and snowball sampling. Considering the inconsistency of the epidemic prevention process in various countries, it may be more difficult to conduct panel study in multiple countries or regions.

## Conclusions

Together, high risk perception and extremely cautious coping behaviors that have been encouraged in the past are not entirely desirable for mental health and should be given special attention. Target policies should be made to achieve an acceptable balance between mental health and physical health at different stages of COVID-19. The findings verified the feasibility of taking risk communication strategies to protect mental health by influencing public risk perception and guiding coping behaviors at different stages.

## Supporting information

S1 Fig Stage changes in coping behaviors during the COVID-19. (DOCX)

S2 Fig Respondents’ concerns and controllability of foreign pandemic in Stage 3 and Stage 4. (DOCX)

S1 Table Subgroup analysis by gender. (DOCX)

S2 Table The reliability and validity test to four stage questionnaires. (DOCX)

S3 Table Demographic characteristics of respondents. (DOCX)

S4 Table Stages changes in risk perceptions and coping behaviors. (DOCX)

S5 Table Risk perception level of respondents in Stage 4. (DOCX)

S6 Table Stage changes in anxiety. (DOCX)

S7 Table Stage changes of risk perceptions, coping behaviors and anxiety levels in different area. (DOCX)

S8 Table Area difference in risk perceptions, coping behaviors and anxiety in each stage and throughout Stage 1 to Stage 3. (DOCX)

S9 Table Factors contributing to high anxiety levels in each stage and throughout Stage 1 to Stage 3. (DOCX)

S10 Table One-way ANOVA analysis: The impact of vaccination on respondents’ perception, behavior, and anxiety levels. (DOCX)

S11 Table Statistics on the number of people vaccinated, vaccination willingness of respondents who hadn’t been vaccinated, and the main reasons for doubts about the vaccine. (DOCX)

S12 Table Socio-demographic characteristics of risk perception in each stage and throughout Stage 1 to Stage 3. (DOCX)

S13 Table Socio-demographic characteristics of coping behaviors in each stage and throughout Stage 1 to Stage 3. (DOCX)

S1 Text Considering both fixed effects and random effects GLMM. (DOCX)

S2 Text Descriptive statistics of all participants. (DOCX)

S3 Text Socio-demographic characteristics of risk perception and coping behaviors. (DOCX)
