# Supplementary material for: Public anxiety through various stages of COVID-19 coping: Evidence from China
Source: PLoS One. 2022 Jun 16;17(6):e0270229. doi: 10.1371/journal.pone.0270229 (PMC9202924; doi:10.1371/journal.pone.0270229)
Supplement: S1 Fig — (DOCX) [file pone.0270229.s001.docx]

**S1 Fig. Stage changes in coping behaviors during the COVID-19**


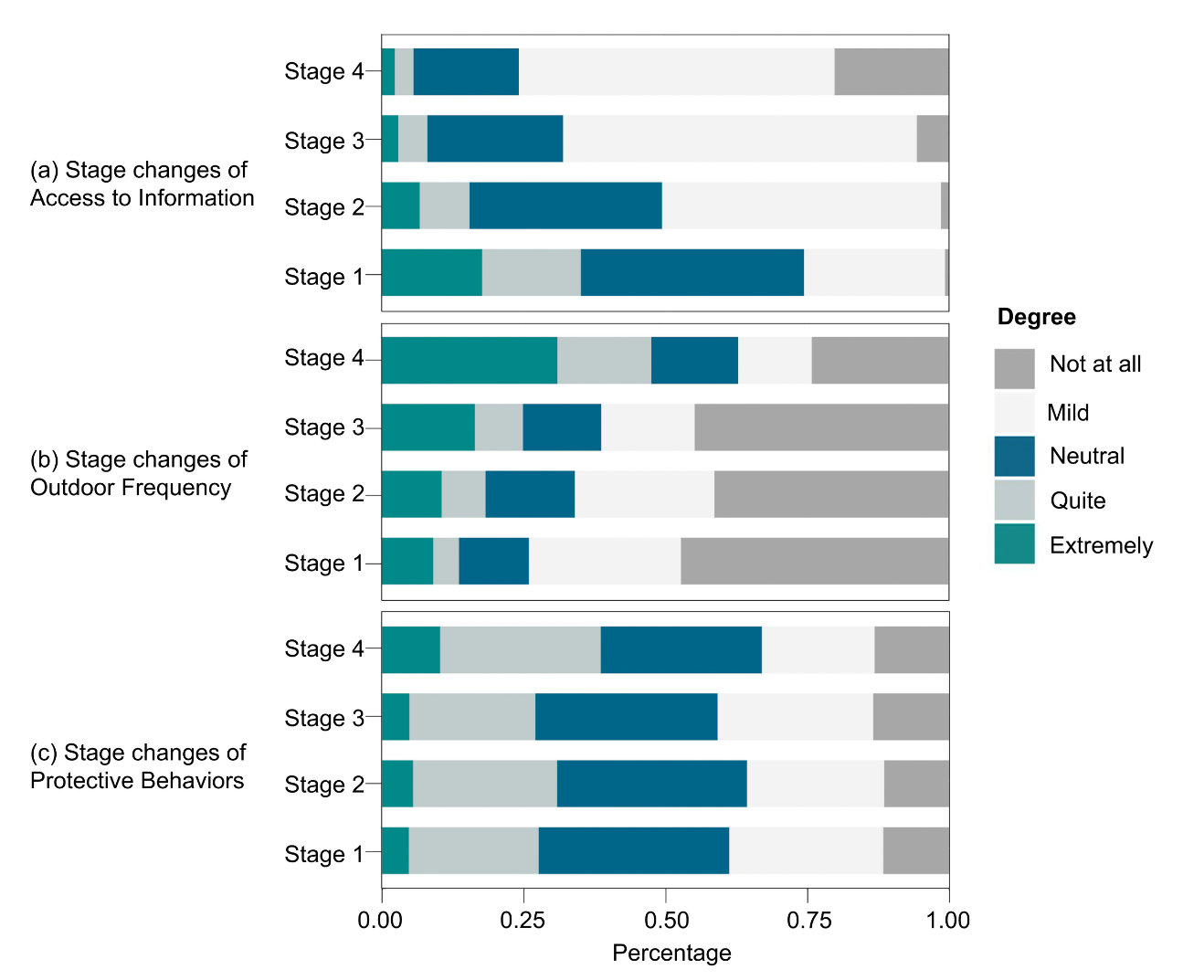


Notes: Access to information represents the frequency of checking information about COVID-19 per day; outdoor activity frequency represents the times of outdoor activity per day; protective behavior degree represents the self-precaution extent at present calculated by the sum of specific protective actions taken.
