# Supplementary material for: Public anxiety through various stages of COVID-19 coping: Evidence from China
Source: PLoS One. 2022 Jun 16;17(6):e0270229. doi: 10.1371/journal.pone.0270229 (PMC9202924; doi:10.1371/journal.pone.0270229)
Supplement: S2 Fig — (DOCX) [file pone.0270229.s002.docx]

**S2 Fig. Respondents' concerns and controllability of foreign pandemic in *Stage 3* and *Stage 4***


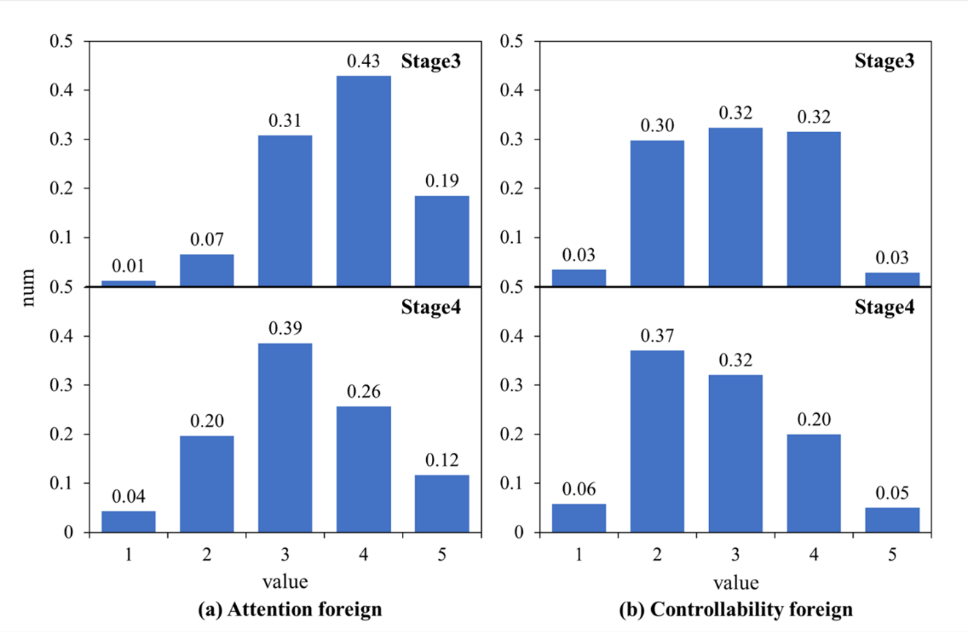


Note: Attention foreign represents the attention levels of the COVID-19 situation in foreign countries and controllability foreign represents the respondents’ belief in the controllability of global COVID-19 crisis. The higher the number, the more they concerned about or believed
