# Supplementary material for: Public anxiety through various stages of COVID-19 coping: Evidence from China
Source: PLoS One. 2022 Jun 16;17(6):e0270229. doi: 10.1371/journal.pone.0270229 (PMC9202924; doi:10.1371/journal.pone.0270229)
Supplement: S1 Table — (DOCX) [file pone.0270229.s003.docx]

**S1 Table. Subgroup analysis by gender**

**S1A Table. The impacts of females' risk perceptions on their own anxiety level in each stage**

| Stage | Risk perception | Posterior mean | Low 95% CI | Up 95% CI | *p* |
| --- | --- | --- | --- | --- | --- |
| Stage 1 | Intercept | 3.46 | 1.56 | 5.30 | 0.001 |
|  | Attention | 0.14 | -0.12 | 0.43 | 0.338 |
|  | Knowledge | -0.12 | -0.40 | 0.14 | 0.414 |
|  | Interference | 0.14 | -0.07 | 0.36 | 0.22 |
|  | Controllability | 0.02 | -0.14 | 0.19 | 0.764 |
|  | Worry about being infected | 0.59 | 0.42 | 0.74 | 0.001 |
|  | Trust | -0.05 | -0.23 | 0.11 | 0.528 |
| Stage 2 | Intercept | 4.64 | 2.83 | 6.40 | 0.001 |
|  | Attention | 0.05 | -0.17 | 0.32 | 0.698 |
|  | Knowledge | -0.29 | -0.56 | 0.01 | 0.058 |
|  | Interference | 0.17 | -0.04 | 0.41 | 0.152 |
|  | Controllability | -0.04 | -0.30 | 0.24 | 0.782 |
|  | Worry about being infected | 0.62 | 0.46 | 0.78 | 0.001 |
|  | Trust | 0.02 | -0.19 | 0.23 | 0.828 |
| Stage 3 | Intercept | 7.04 | 4.70 | 9.55 | 0.001 |
|  | Attention | 0.19 | -0.08 | 0.48 | 0.182 |
|  | Knowledge | -0.39 | -0.80 | 0.02 | 0.066 |
|  | Interference | 0.19 | -0.06 | 0.48 | 0.188 |
|  | Controllability | -0.18 | -0.61 | 0.19 | 0.382 |
|  | Worry about being infected | 0.39 | 0.18 | 0.59 | 0.001 |
|  | Trust | -0.15 | -0.42 | 0.18 | 0.34 |
| Stage 4 | Intercept | 4.63 | 2.60 | 6.85 | 0.001 |
|  | Attention domestic | -0.28 | -0.66 | 0.05 | 0.106 |
|  | Attention global | 0.24 | -0.01 | 0.54 | 0.074 |
|  | Controllability domestic | 0.04 | -0.34 | 0.45 | 0.864 |
|  | Controllability global | -0.53 | -0.77 | -0.25 | 0.001 |
|  | Worry about being infected | 0.57 | 0.35 | 0.79 | 0.001 |
|  | Worry global pandemic | 0.45 | 0.15 | 0.76 | 0.002 |
|  | Interference | -0.02 | -0.30 | 0.28 | 0.894 |
|  | Vaccine trust | 0.11 | -0.18 | 0.41 | 0.46 |

**S1B Table. The impacts of males' risk perceptions on their own anxiety level in each stage**

| Stage | Risk perception | Posterior mean | Low 95% CI | Up 95% CI | *p* |
| --- | --- | --- | --- | --- | --- |
| Stage 1 | Intercept | 2.35 | 0.82 | 4.21 | 0.01 |
|  | Attention | 0.03 | -0.24 | 0.25 | 0.806 |
|  | Knowledge | 0.01 | -0.27 | 0.30 | 0.948 |
|  | Interference | 0.05 | -0.18 | 0.28 | 0.694 |
|  | Controllability | 0.17 | 0.01 | 0.33 | 0.036 |
|  | Worry about being infected | 0.44 | 0.26 | 0.61 | 0.001 |
|  | Trust | 0.11 | -0.07 | 0.29 | 0.232 |
| Stage 2 | Intercept | 3.26 | 1.26 | 5.25 | 0.002 |
|  | Attention | 0.15 | -0.14 | 0.45 | 0.312 |
|  | Knowledge | 0.03 | -0.27 | 0.34 | 0.846 |
|  | Interference | 0.20 | -0.05 | 0.47 | 0.146 |
|  | Controllability | -0.09 | -0.38 | 0.20 | 0.554 |
|  | Worry about being infected | 0.65 | 0.47 | 0.83 | 0.001 |
|  | Trust | -0.25 | -0.53 | -0.01 | 0.066 |
| Stage 3 | Intercept | 5.78 | 3.07 | 8.54 | 0.001 |
|  | Attention | 0.26 | -0.04 | 0.57 | 0.082 |
|  | Knowledge | 0.11 | -0.32 | 0.51 | 0.612 |
|  | Interference | 0.08 | -0.21 | 0.40 | 0.634 |
|  | Controllability | -0.63 | -1.06 | -0.21 | 0.004 |
|  | Worry about being infected | 0.42 | 0.19 | 0.63 | 0.002 |
|  | Trust | 0.01 | -0.35 | 0.41 | 0.988 |
| Stage 4 | Intercept | 3.50 | 1.03 | 5.51 | 0.004 |
|  | Attention domestic | 0.31 | -0.06 | 0.67 | 0.082 |
|  | Attention global | 0.03 | -0.29 | 0.36 | 0.8 |
|  | Controllability domestic | -0.24 | -0.65 | 0.20 | 0.294 |
|  | Controllability global | 0.16 | -0.15 | 0.42 | 0.254 |
|  | Worry about being infected | 0.41 | 0.19 | 0.63 | 0.001 |
|  | Worry global pandemic | 0.51 | 0.17 | 0.85 | 0.002 |
|  | Interference | -0.14 | -0.48 | 0.22 | 0.422 |
|  | Vaccine trust | -0.27 | -0.64 | 0.09 | 0.14 |

**S1C Table. The impacts of females' coping behaviors on their own anxiety level in each stage**

| Stage | Coping behavior | Posterior mean | Low 95% CI | Up 95% CI | *p* |
| --- | --- | --- | --- | --- | --- |
| Stage 1 | Intercept | 4.60 | 3.54 | 5.65 | 0.001 |
|  | Access to information | 0.24 | 0.06 | 0.41 | 0.008 |
|  | Outdoor activity | 0.04 | -0.05 | 0.14 | 0.402 |
|  | Protective behavior | 0.06 | -0.03 | 0.15 | 0.22 |
|  | Community closed | 0.42 | -0.07 | 0.89 | 0.11 |
| Stage 2 | Intercept | 4.79 | 3.67 | 5.87 | 0.001 |
|  | Access to information | 0.18 | -0.02 | 0.40 | 0.098 |
|  | Outdoor activity | 0.08 | -0.04 | 0.19 | 0.174 |
|  | Protective behavior | 0.11 | 0.02 | 0.20 | 0.024 |
|  | Community closed | 0.03 | -0.55 | 0.56 | 0.914 |
| Stage 3 | Intercept | 4.60 | 3.45 | 5.78 | 0.001 |
|  | Access to information | 0.20 | -0.11 | 0.50 | 0.21 |
|  | Outdoor activity | 0.08 | -0.02 | 0.18 | 0.11 |
|  | Protective behavior | 0.15 | 0.05 | 0.25 | 0.004 |
|  | Community closed | -0.36 | -2.16 | 1.23 | 0.684 |
| Stage 4 | Intercept | 3.08 | 0.44 | 5.89 | 0.038 |
|  | Access to information | 0.26 | -0.02 | 0.53 | 0.07 |
|  | Outdoor activity | 0.58 | 0.14 | 0.94 | 0.006 |
|  | Protective behavior | 0.13 | 0.03 | 0.23 | 0.016 |
|  | Vaccination | 0.62 | -0.38 | 1.93 | 0.28 |

**S1D Table. The impacts of males' coping behaviors on their own anxiety level in each stage**

| Stage | Coping behavior | Posterior mean | Low 95% CI | Up 95% CI | *p* |
| --- | --- | --- | --- | --- | --- |
| Stage 1 | Intercept | 5.28 | 4.46 | 6.29 | 0.001 |
|  | Access to information | 0.12 | -0.05 | 0.31 | 0.214 |
|  | Outdoor activity | -0.09 | -0.17 | -0.02 | 0.016 |
|  | Protective behavior | 0.01 | -0.07 | 0.09 | 0.854 |
|  | Community closed | 0.42 | -0.07 | 0.91 | 0.102 |
| Stage 2 | Intercept | 3.00 | 1.76 | 4.14 | 0.001 |
|  | Access to information | 0.39 | 0.12 | 0.63 | 0.002 |
|  | Outdoor activity | 0.04 | -0.04 | 0.12 | 0.36 |
|  | Protective behavior | 0.17 | 0.08 | 0.27 | 0.001 |
|  | Community closed | 0.27 | -0.49 | 0.93 | 0.484 |
| Stage 3 | Intercept | 3.29 | 1.94 | 4.64 | 0.001 |
|  | Access to information | 0.24 | -0.06 | 0.61 | 0.138 |
|  | Outdoor activity | 0.15 | 0.05 | 0.24 | 0.001 |
|  | Protective behavior | 0.12 | 0.00 | 0.24 | 0.028 |
|  | Community closed | -0.73 | -2.37 | 0.92 | 0.4 |
| Stage 4 | Intercept | 4.63 | 2.30 | 7.15 | 0.001 |
|  | Access to information | 0.58 | 0.24 | 0.85 | 0.001 |
|  | Outdoor activity | 0.04 | -0.34 | 0.45 | 0.842 |
|  | Protective behavior | 0.11 | 0.02 | 0.23 | 0.044 |
|  | Vaccination | -0.47 | -1.54 | 0.55 | 0.396 |
