# Supplementary material for: Public anxiety through various stages of COVID-19 coping: Evidence from China
Source: PLoS One. 2022 Jun 16;17(6):e0270229. doi: 10.1371/journal.pone.0270229 (PMC9202924; doi:10.1371/journal.pone.0270229)
Supplement: S2 Table — (DOCX) [file pone.0270229.s004.docx]

**S2 Table. The reliability and validity test to four stage questionnaires**

| Analytical method | *Stage 1* | *Stage 2* | *Stage 3* | *Stage 4* |
| --- | --- | --- | --- | --- |
| Cronbach's α | 0.83 | 0.71 | 0.71 | 0.82 |
| Kaiser-Meyer-Olkin | 0.79 | 0.76 | 0.74 | 0.92 |
| Bartlett’s Test of Sphericity | p<0.05 | p<0.05 | p<0.05 | p<0.05 |
