# Supplementary material for: Public anxiety through various stages of COVID-19 coping: Evidence from China
Source: PLoS One. 2022 Jun 16;17(6):e0270229. doi: 10.1371/journal.pone.0270229 (PMC9202924; doi:10.1371/journal.pone.0270229)
Supplement: S3 Table — (DOCX) [file pone.0270229.s005.docx]

**S3 Table. Demographic characteristics of respondents**

| Variables | Class | Count | Percent (%) |
| --- | --- | --- | --- |
| Gender | Male | 2573 | 43.0% |
|  | Female | 3410 | 57.0% |
|  | Total | 5983 | |
| Education | Primary school | 57 | 1.0% |
|  | Middle school | 441 | 7.4% |
|  | High school | 592 | 9.9% |
|  | Polytechnic College | 516 | 8.6% |
|  | Bachelor | 2644 | 44.2% |
|  | Master | 1407 | 23.5% |
|  | PhD | 326 | 5.4% |
| Age | <18 | 187 | 3.1% |
|  | 18~25 | 3085 | 51.6% |
|  | 26~30 | 755 | 12.6% |
|  | 31~40 | 782 | 13.1% |
|  | 41~50 | 816 | 13.6% |
|  | 51~60 | 290 | 4.8% |
|  | >60 | 68 | 1.1% |
| Occupation | Student | 2714 | 45.4% |
|  | Labor worker | 387 | 6.5% |
|  | Farmer | 117 | 2.0% |
|  | Medical worker | 114 | 1.9% |
|  | Teacher | 571 | 9.5% |
|  | Official | 209 | 3.5% |
|  | Businessman | 209 | 3.5% |
|  | Others | 1662 | 27.8% |
| Stage | *Stage 1* | 2087 | 34.9% |
|  | *Stage 2* | 1731 | 28.9% |
|  | *Stage 3* | 1129 | 18.9% |
|  | *Stage 4* | 1036 | 17.3% |
