# Supplementary material for: Public anxiety through various stages of COVID-19 coping: Evidence from China
Source: PLoS One. 2022 Jun 16;17(6):e0270229. doi: 10.1371/journal.pone.0270229 (PMC9202924; doi:10.1371/journal.pone.0270229)
Supplement: S4 Table — (DOCX) [file pone.0270229.s006.docx]

**S4 Table. Stages changes in risk perceptions and coping behaviors**

**S4A Table. Compared with stage 1, the changes in risk perception and response behavior of respondents in other stages**

| Variables | Post. mean | l-95% CI | u-95% CI | p |
| --- | --- | --- | --- | --- |
| **Worry about being infected** | | |  |  |
| Stage 2 | -0.46 | -0.53 | -0.38 | 0.001 |
| Stage 3 | -1.11 | -1.19 | -1.02 | 0.001 |
| Stage 4 | -0.59 | -0.68 | -0.5 | 0.001 |
| **Attention** |  |  |  |  |
| Stage 2 | -0.17 | -0.22 | -0.11 | 0.001 |
| Stage 3 | -0.87 | -0.94 | -0.81 | 0.001 |
| Stage 4 | -0.6 | -0.66 | -0.54 | 0.001 |
| **Controllability** | |  |  |  |
| Stage 2 | 0.96 | 0.91 | 1.02 | 0.001 |
| Stage 3 | 1.28 | 1.22 | 1.35 | 0.001 |
| Stage 4 | 1.22 | 1.15 | 1.27 | 0.001 |
| **Interference** | |  |  |  |
| Stage 2 | -0.01 | -0.06 | 0.05 | 0.842 |
| Stage 3 | -0.34 | -0.41 | -0.28 | 0.001 |
| Stage 4 | -0.59 | -0.65 | -0.52 | 0.001 |
| **Precautions** |  |  |  |  |
| Stage 2 | -0.28 | -0.33 | -0.24 | 0.001 |
| Stage 3 | -0.77 | -0.82 | -0.72 | 0.001 |
| **Trust** |  |  |  |  |
| Stage 2 | 0.15 | 0.09 | 0.21 | 0.001 |
| Stage 3 | 0.48 | 0.41 | 0.54 | 0.001 |
| **Knowledge** |  |  |  |  |
| Stage 2 | 0.03 | -0.01 | 0.07 | 0.132 |
| Stage 3 | -0.10 | -0.15 | -0.05 | 0.001 |
| **K. Symptoms** | |  |  |  |
| Stage 2 | 0.07 | 0.02 | 0.11 | 0.008 |
| Stage 3 | -0.02 | -0.08 | 0.03 | 0.362 |
| **K. Transmissions** | |  |  |  |
| Stage 2 | -0.01 | -0.06 | 0.03 | 0.588 |
| Stage 3 | -0.10 | -0.16 | -0.05 | 0.001 |
| **K. Treatment** | |  |  |  |
| Stage 2 | 0.19 | 0.12 | 0.25 | 0.001 |
| Stage 3 | 0.26 | 0.18 | 0.33 | 0.001 |
| **K. Source** |  |  |  |  |
| Stage 2 | -0.10 | -0.16 | -0.04 | 0.001 |
| Stage 3 | -0.50 | -0.56 | -0.43 | 0.001 |
| **Access to information** | |  |  |  |
| Stage 2 | -0.57 | -0.63 | -0.51 | 0.001 |
| Stage 3 | -0.90 | -0.97 | -0.83 | 0.001 |
| Stage 4 | -1.15 | -1.22 | -1.08 | 0.001 |
| **Outdoor activity** | |  |  |  |
| Stage 2 | 0.22 | 0.06 | 0.38 | 0.001 |
| Stage 3 | 2.00 | 1.81 | 2.17 | 0.004 |
| Stage 4 | 3.39 | 3.21 | 3.56 | 0.001 |
| **Protective behavior** | |  |  |  |
| Stage 2 | 0.11 | -0.04 | 0.27 | 0.168 |
| Stage 3 | -0.13 | -0.29 | 0.04 | 0.154 |
| Stage 4 | 0.30 | 0.11 | 0.47 | 0.001 |

Note: 95% CI means 95% Highest posterior density (HPD) interval

**S4B Table. Testing whether the mean value of respondents' risk perceptions and coping behaviors in each stage were significantly different through Welch and Brown-Forsythe test**

| Variables | Test | p |
| --- | --- | --- |
| **Risk perceptions** |  |  |
| Attention | Welch | <0.001 |
|  | Brown-Forsythe(B) | <0.001 |
| Interference | Welch | <0.001 |
|  | Brown-Forsythe(B) | <0.001 |
| Worry be infected | Welch | <0.001 |
|  | Brown-Forsythe(B) | <0.001 |
| Trust | Welch | <0.001 |
|  | Brown-Forsythe(B) | <0.001 |
| Controllability | Welch | <0.001 |
|  | Brown-Forsythe(B) | <0.001 |
| Precautions | Welch | <0.001 |
|  | Brown-Forsythe(B) | <0.001 |
| ***Knowledge*** |  |  |
| (a) Infection symptoms | Welch | 0.001 |
|  | Brown-Forsythe(B) | 0.001 |
| (b) Transmission | Welch | <0.001 |
|  | Brown-Forsythe(B) | <0.001 |
| (c) Treatment | Welch | <0.001 |
|  | Brown-Forsythe(B) | <0.001 |
| (d) Virus source | Welch | <0.001 |
|  | Brown-Forsythe(B) | <0.001 |
| **Coping behaviors** |  |  |
| Access to information | Welch | <0.001 |
|  | Brown-Forsythe(B) | <0.001 |
| Outdoor activity | Welch | <0.001 |
|  | Brown-Forsythe(B) | <0.001 |
| Protective behavior | Welch | <0.001 |
|  | Brown-Forsythe(B) | <0.001 |

**S4C Table. Mean value of respondents' risk perceptions and coping behaviors in each stage**

| Mean value | *Stage 1* | *Stage 2* | *Stage 3* | *Stage 4* |
| --- | --- | --- | --- | --- |
| **Risk perceptions** |  |  |  |  |
| Attention | 4.64 | 4.47 | 3.77 | 4.03 |
| Interference | 4.15 | 4.15 | 3.81 | 3.56 |
| Controllability | 3.28 | 4.25 | 4.57 | 4.50 |
| Worry be infected | 3.86 | 3.40 | 2.75 | 3.27 |
| Trust | 3.97 | 4.12 | 4.44 |  |
| ***Knowledge*** |  |  |  |  |
| (a) Infection symptoms | 4.06 | 4.13 | 4.04 |  |
| (b) Transmission | 4.15 | 4.14 | 4.04 |  |
| (c) Treatment | 3.16 | 3.35 | 3.42 |  |
| (d) Virus source | 3.81 | 3.72 | 3.32 |  |
| **Coping behaviors** |  |  |  |  |
| Access to information | 3.28 | 2.71 | 2.38 | 2.12 |
| Outdoor activity | 6.23 | 6.45 | 8.23 | 9.62 |
| Protective behavior | 6.13 | 6.24 | 6.01 | 6.43 |
