# Supplementary material for: Public anxiety through various stages of COVID-19 coping: Evidence from China
Source: PLoS One. 2022 Jun 16;17(6):e0270229. doi: 10.1371/journal.pone.0270229 (PMC9202924; doi:10.1371/journal.pone.0270229)
Supplement: S5 Table — (DOCX) [file pone.0270229.s007.docx]

**S5 Table. Risk perception level of respondents in Stage 4**

|  | Not at all | Not quite | Commonly | Quite | Extremely |
| --- | --- | --- | --- | --- | --- |
| **Attention** | | | | | |
| Domestic | 11(1.1%) | 51(4.9%) | 176(17.0%) | 456(44.0%) | 342(33.0%) |
| Foreign | 45(4.3%) | 204(19.7%) | 400(38.6%) | 266(25.7%) | 121(11.7%) |
| **Controllability** | | | | | |
| Domestic | 1(0.1%) | 11(1.1%) | 34(3.3%) | 410(39.6%) | 580(56.0%) |
| Foreign | 60(5.8%) | 384(37.1%) | 333(32.1%) | 207(20.0%) | 52(5.0%) |
| **Worry** | | | | | |
| Being infected | 112(10.8%) | 192(18.5%) | 260(25.1%) | 251(24.2%) | 221(21.3%) |
| Cold chain food | 29(2.8%) | 33(3.2%) | 127(12.3%) | 494(47.7%) | 353(34.1%) |
| Imported goods | 33(3.2%) | 44(4.2%) | 138(13.3%) | 454(43.8%) | 367(35.4%) |
| Study abroad | 169(16.3%) | 132(12.7%) | 254(24.5%) | 278(26.8%) | 203(19.6%) |
| Incomes | 90(8.7%) | 141(13.6%) | 268(25.9%) | 333(32.1%) | 204(19.7%) |
| Reunite | 73(7.0%) | 81(7.8%) | 190(18.3%) | 382(36.9%) | 310(29.9%) |
| **Interference** | 26(2.5%) | 75(7.2%) | 358(34.6%) | 444(42.9%) | 133(12.8%) |
| **Vaccine trust** | 9(0.9%) | 31(3.0%) | 256(24.7%) | 481(46.4%) | 259(25.0%) |
