# Supplementary material for: Public anxiety through various stages of COVID-19 coping: Evidence from China
Source: PLoS One. 2022 Jun 16;17(6):e0270229. doi: 10.1371/journal.pone.0270229 (PMC9202924; doi:10.1371/journal.pone.0270229)
Supplement: S6 Table — (DOCX) [file pone.0270229.s008.docx]

**S6 Table. Stage changes in anxiety**

| Reference | anxiety | Post. mean | l-95% CI | u-95% CI | p |
| --- | --- | --- | --- | --- | --- |
| *Stage 1* | Stage 2 | 0.087 | -0.118 | 0.239 | 0.334 |
|  | Stage 3 | 0.264 | 0.070 | 0.491 | 0.014 |
|  | Stage 4 | 0.295 | 0.098 | 0.512 | 0.008 |
| *Stage 2* | Stage 3 | 0.180 | -0.047 | 0.387 | 0.126 |
|  | Stage 4 | 0.204 | -0.031 | 0.434 | 0.092 |
| *Stage 3* | Stage 4 | 0.027 | -0.209 | 0.289 | 0.848 |

Note: 95% CI means 95% Highest posterior density (HPD) interval; 95% CI means 95% Highest posterior density (HPD) interval; L-95% CI and U-95% CI represent the upper and lower limits of 95% CI respectively; p: MCMC p-values, the probability from linear mixed models using Markov Chain Monte Carlo (MCMC) methods; *p<0.05, **p<0.01, ***p<0.001.
