# Supplementary material for: Public anxiety through various stages of COVID-19 coping: Evidence from China
Source: PLoS One. 2022 Jun 16;17(6):e0270229. doi: 10.1371/journal.pone.0270229 (PMC9202924; doi:10.1371/journal.pone.0270229)
Supplement: S7 Table — (DOCX) [file pone.0270229.s009.docx]

**S7 Table. Stage changes of risk perceptions, coping behaviors and anxiety levels in different area**

**S7A Table. Mean value of perception, behavior and anxiety level in different area at each stage**

**S7B Table. Respondents’ average risk perception level of global pandemic and their trust in vaccines in Stage 4**

| Area | Eastern | Northeast | Northern | Northwest | South Central | Southwest |
| --- | --- | --- | --- | --- | --- | --- |
| Attention foreign | 4.04 | 4.12 | 3.95 | 4.04 | 3.99 | 3.98 |
| Controllability foreign | 2.76 | 3.03 | 3.07 | 2.5 | 2.81 | 2.84 |
| Vaccine trust | 3.91 | 3.96 | 4.07 | 3.88 | 3.86 | 3.85 |
| ***Worry about global pandemic*** | | | | | | |
| (a) cold food | 4.08 | 4.16 | 4.11 | 4.04 | 3.91 | 4.06 |
| (b) imported goods | 4.03 | 4.15 | 4.09 | 4.04 | 3.88 | 4.12 |
| (c) study abroad | 3.15 | 3.16 | 3.31 | 3.42 | 3.33 | 3.31 |
| (d) incomes | 3.31 | 3.58 | 3.72 | 3.67 | 3.38 | 3.56 |
| (e) reunite | 3.65 | 3.64 | 4.05 | 3.83 | 4.02 | 3.9 |
