# Supplementary material for: Public anxiety through various stages of COVID-19 coping: Evidence from China
Source: PLoS One. 2022 Jun 16;17(6):e0270229. doi: 10.1371/journal.pone.0270229 (PMC9202924; doi:10.1371/journal.pone.0270229)
Supplement: S8 Table — (DOCX) [file pone.0270229.s010.docx]

**S8 Table. Area difference in risk perceptions, coping behaviors and anxiety in each stage and throughout Stage 1 to Stage3**

**S8A Table. Area difference in anxiety in each stage**

|  | *Stage 1* |  |  |  | *Stage 2* |  |  |  |
| --- | --- | --- | --- | --- | --- | --- | --- | --- |
|  | Post. mean | l-95% CI | u-95% CI | p | Post. mean | l-95% CI | u-95% CI | p |
| Gender | 0.93 | 0.69 | 1.16 | 0.00 | 0.90 | 0.64 | 1.18 | 0.00 |
| Education | -0.31 | -0.41 | -0.21 | 0.00 | -0.27 | -0.37 | -0.15 | 0.00 |
| Age | 0.03 | -0.07 | 0.14 | 0.55 | 0.18 | 0.04 | 0.29 | 0.01 |
| Occupation | 0.00 | -0.03 | 0.03 | 0.90 | 0.01 | -0.03 | 0.04 | 0.69 |
| Northeast | -0.07 | -0.51 | 0.38 | 0.74 | 0.21 | -0.29 | 0.76 | 0.47 |
| Northern | -0.31 | -0.74 | 0.10 | 0.16 | -0.21 | -0.65 | 0.29 | 0.41 |
| Northwest | 0.67 | 0.18 | 1.16 | 0.01 | 0.78 | 0.16 | 1.36 | 0.01 |
| South Central | -0.29 | -0.69 | 0.10 | 0.14 | -0.06 | -0.52 | 0.44 | 0.80 |
| Southwest | -0.04 | -0.46 | 0.41 | 0.88 | 0.00 | -0.52 | 0.47 | 0.99 |
|  | *Stage 3* |  |  |  | *Stage 4* |  |  |  |
|  | Post. mean | l-95% CI | u-95% CI | p | Post. mean | l-95% CI | u-95% CI | p |
| Gender | 0.81 | 0.47 | 1.17 | 0.00 | 1.07 | 0.74 | 1.43 | 0.00 |
| Education | -0.33 | -0.47 | -0.18 | 0.00 | -0.40 | -0.55 | -0.27 | 0.00 |
| Age | 0.17 | 0.04 | 0.33 | 0.02 | 0.08 | -0.07 | 0.23 | 0.27 |
| Occupation | -0.03 | -0.08 | 0.01 | 0.18 | -0.01 | -0.08 | 0.05 | 0.72 |
| Northeast | 0.10 | -0.66 | 0.92 | 0.86 | 0.69 | 0.05 | 1.48 | 0.054 |
| Northern | -0.07 | -0.69 | 0.51 | 0.79 | -0.07 | -0.69 | 0.53 | 0.80 |
| Northwest | 0.82 | 0.08 | 1.64 | 0.04 | 0.25 | -0.90 | 1.46 | 0.65 |
| South Central | -0.05 | -0.58 | 0.54 | 0.86 | 0.00 | -0.66 | 0.58 | 1.00 |
| Southwest | -0.25 | -0.84 | 0.38 | 0.44 | -0.47 | -1.03 | 0.16 | 0.12 |

Note: 95% CI means 95% Highest posterior density (HPD) interval

**S8B Table. Area difference of risk perception, coping behavior and anxiety level throughout Stage 1 to Stage3.**

| Variables | Area | Post. mean | l-95% CI | u-95% CI | p | sig. |
| --- | --- | --- | --- | --- | --- | --- |
| **Risk perception** | | | | | | |
| Attention | Northeast China | -0.034 | -0.233 | 0.194 | 0.760 |  |
|  | Northern China | 0.012 | -0.163 | 0.199 | 0.904 |  |
|  | Northwest China | 0.153 | -0.070 | 0.409 | 0.200 |  |
|  | Southwest China | 0.220 | 0.023 | 0.411 | 0.024 | * |
|  | Central-south China | 0.110 | -0.056 | 0.284 | 0.228 |  |
| Controllability | Northeast China | 0.103 | 0.000 | 0.201 | 0.048 | * |
|  | Northern China | 0.045 | -0.049 | 0.124 | 0.322 |  |
|  | Northwest China | 0.041 | -0.063 | 0.146 | 0.450 |  |
|  | Southwest China | 0.069 | -0.015 | 0.146 | 0.114 |  |
|  | Central-south China | -0.041 | -0.131 | 0.039 | 0.364 |  |
| Understand | Northeast China | -0.047 | -0.116 | 0.037 | 0.238 |  |
|  | Northern China | -0.066 | -0.132 | 0.008 | 0.070 |  |
|  | Northwest China | 0.023 | -0.061 | 0.105 | 0.576 |  |
|  | Southwest China | -0.037 | -0.109 | 0.027 | 0.276 |  |
|  | Central-south China | 0.004 | -0.056 | 0.064 | 0.886 |  |
| Worry | Northeast China | -0.041 | -0.203 | 0.103 | 0.556 |  |
|  | Northern China | 0.032 | -0.098 | 0.159 | 0.604 |  |
|  | Northwest China | 0.180 | 0.015 | 0.338 | 0.028 | * |
|  | Southwest China | 0.142 | 0.000 | 0.268 | 0.042 | * |
|  | Central-south China | 0.107 | -0.015 | 0.219 | 0.082 |  |
| Trust | Northeast China | -0.320 | -0.553 | -0.092 | 0.002 | ** |
|  | Northern China | -0.139 | -0.346 | 0.039 | 0.142 |  |
|  | Northwest China | -0.132 | -0.383 | 0.107 | 0.268 |  |
|  | Southwest China | -0.154 | -0.341 | 0.038 | 0.082 |  |
|  | Central-south China | -0.504 | -0.758 | -0.283 | 0.001 | *** |
| Interference | Northeast China | -0.310 | -0.405 | -0.213 | 0.001 | *** |
|  | Northern China | -0.094 | -0.169 | -0.008 | 0.020 | * |
|  | Northwest China | 0.148 | 0.053 | 0.264 | 0.006 | ** |
|  | Southwest China | -0.017 | -0.108 | 0.063 | 0.690 |  |
|  | Central-south China | 0.034 | -0.040 | 0.111 | 0.406 |  |
| **Coping behavior** | | | | | | |
| Precaution extent | Northeast China | -0.072 | -0.148 | 0.008 | 0.068 |  |
|  | Northern China | -0.029 | -0.099 | 0.037 | 0.420 |  |
|  | Northwest China | -0.134 | -0.223 | -0.048 | 0.010 | ** |
|  | Southwest China | -0.036 | -0.102 | 0.035 | 0.318 |  |
|  | Central-south China | -0.004 | -0.069 | 0.056 | 0.918 |  |
| Protective behavior | Northeast China | -0.711 | -0.960 | -0.479 | 0.001 | *** |
|  | Northern China | -0.269 | -0.495 | -0.068 | 0.022 | * |
|  | Northwest China | -0.009 | -0.300 | 0.242 | 0.976 |  |
|  | Southwest China | 0.136 | -0.094 | 0.340 | 0.216 |  |
|  | Central-south China | 0.034 | -0.183 | 0.236 | 0.762 |  |
| Outdoor activity | Northeast China | -0.348 | -0.615 | -0.114 | 0.002 | ** |
|  | Northern China | -0.269 | -0.489 | -0.073 | 0.008 | ** |
|  | Northwest China | 0.355 | 0.077 | 0.599 | 0.008 | ** |
|  | Southwest China | 0.547 | 0.311 | 0.754 | 0.001 | *** |
|  | Central-south China | -0.155 | -0.359 | 0.056 | 0.154 |  |
| Access to information | Northeast China | -0.064 | -0.168 | 0.040 | 0.252 |  |
|  | Northern China | 0.052 | -0.039 | 0.141 | 0.268 |  |
|  | Northwest China | 0.059 | -0.050 | 0.177 | 0.292 |  |
|  | Southwest China | 0.096 | 0.001 | 0.185 | 0.038 | * |
|  | Central-south China | 0.092 | 0.011 | 0.182 | 0.042 | * |
| **Crown-Crisp index phobic anxiety** | | | | | | |
| Anxiety | Northeast China | 0.106 | -0.205 | 0.435 | 0.532 |  |
|  | Northern China | -0.225 | -0.482 | 0.047 | 0.094 |  |
|  | Northwest China | 0.756 | 0.393 | 1.100 | 0.001 |  |
|  | Southwest China | -0.069 | -0.355 | 0.217 | 0.616 |  |
|  | Central-south China | -0.200 | -0.494 | 0.038 | 0.140 |  |

Note: 95% CI means 95% Highest posterior density (HPD) interval; Other areas were compared with Northern China; L-95% CI and U-95% CI represent the upper and lower limits of 95% CI respectively; p: MCMC p-values, the probability from linear mixed models using Markov Chain Monte Carlo (MCMC) methods; *p<0.05, **p<0.01, ***p<0.001.
