# Supplementary material for: Public anxiety through various stages of COVID-19 coping: Evidence from China
Source: PLoS One. 2022 Jun 16;17(6):e0270229. doi: 10.1371/journal.pone.0270229 (PMC9202924; doi:10.1371/journal.pone.0270229)
Supplement: S9 Table — (DOCX) [file pone.0270229.s011.docx]

**S9 Table. Factors contributing to high anxiety levels in each stage and throughout Stage 1 to Stage3**

**S9A Table. Factors contributing to high anxiety levels in Stage 1, 2, 3**

Note: 95% CI means 95% Highest posterior density (HPD) interval; L-95% CI and U-95% CI represent the upper and lower limits of 95% CI respectively; p: MCMC p-values, the probability from linear mixed models using Markov Chain Monte Carlo (MCMC) methods; *p<0.05, **p<0.01, ***p<0.001.

**S9B Table. Factors contributing to high anxiety levels from Stage 1 to Stage 3**

| Factors | Variables | Post. mean | l-95% CI | u-95% CI | p |
| --- | --- | --- | --- | --- | --- |
| Anxiety-individual | Age | 0.100 | 0.035 | 0.164 | 0.001 |
|  | Gender | 0.901 | 0.740 | 1.058 | 0.001 |
|  | Education | -0.308 | -0.374 | -0.235 | 0.001 |
|  | Closing community | 0.196 | -0.071 | 0.452 | 0.140 |
|  | Occupation | -0.003 | -0.025 | 0.016 | 0.768 |
|  | Area | 0.100 | 0.035 | 0.177 | 0.004 |
| Anxiety-perception | Attention | 0.134 | 0.023 | 0.254 | 0.020 |
|  | Controllability | -0.013 | -0.105 | 0.079 | 0.804 |
|  | Knowledge | -0.112 | -0.251 | 0.018 | 0.100 |
|  | Worry about being infected | 0.558 | 0.485 | 0.628 | 0.001 |
|  | Trust | -0.083 | -0.170 | 0.008 | 0.080 |
|  | Interference | 0.106 | 0.006 | 0.210 | 0.038 |
| Anxiety-behavior | Protective behavior | 0.120 | 0.080 | 0.158 | 0.001 |
|  | Access to information | 0.207 | 0.116 | 0.306 | 0.001 |
|  | Outdoor activity | 0.020 | -0.011 | 0.053 | 0.242 |
|  | Precautions | 0.153 | 0.032 | 0.266 | 0.006 |

Note: 95% CI means 95% Highest posterior density (HPD) interval; L-95% CI and U-95% CI represent the upper and lower limits of 95% CI respectively; p: MCMC p-values, the probability from linear mixed models using Markov Chain Monte Carlo (MCMC) methods; *p<0.05, **p<0.01, ***p<0.001.

**S9C Table. Factors contributing to high anxiety levels in Stage 4**

|  | Post. mean | l-95% CI | u-95% CI | p |
| --- | --- | --- | --- | --- |
| **Individual factors** |  |  |  |  |
| Gender | 1.095 | 0.737 | 1.452 | 0.001 |
| Education | -0.418 | -0.560 | -0.280 | 0.001 |
| Age | 0.067 | -0.063 | 0.221 | 0.384 |
| Occupation | -0.011 | -0.074 | 0.060 | 0.750 |
| Area | -0.056 | -0.160 | 0.031 | 0.264 |
| **Worry** |  |  |  |  |
| Worry about being infected | 0.450 | 0.308 | 0.612 | 0.001 |
| Worry incomes | 0.385 | 0.216 | 0.601 | 0.001 |
| Worry reunite | -0.005 | -0.159 | 0.166 | 0.984 |
| Worry study abroad | -0.022 | -0.179 | 0.137 | 0.782 |
| Worry Cold chain food | 0.067 | -0.302 | 0.380 | 0.720 |
| Worry imported goods | 0.092 | -0.231 | 0.417 | 0.578 |
| **Other risk perceptions** |  |  |  |  |
| Attention domestic | 0.047 | -0.214 | 0.262 | 0.706 |
| Attention foreign | 0.090 | -0.122 | 0.278 | 0.402 |
| Controllability domestic | -0.068 | -0.363 | 0.245 | 0.676 |
| Controllability foreign | -0.155 | -0.325 | 0.044 | 0.116 |
| Add worry global | 0.540 | 0.310 | 0.783 | 0.001 |
| Interference | -0.123 | -0.349 | 0.070 | 0.256 |
| Vaccine trust | -0.142 | -0.366 | 0.090 | 0.250 |
| **Coping Behaviors** |  |  |  |  |
| Protective behavior | 0.147 | 0.071 | 0.214 | 0.001 |
| Outdoor activity | 0.225 | -0.084 | 0.508 | 0.148 |
| Access to information | 0.361 | 0.173 | 0.607 | 0.001 |
| **Spatial difference** |  |  |  |  |
| Gender | 1.070 | 0.744 | 1.427 | 0.001 |
| Education | -0.404 | -0.548 | -0.273 | 0.001 |
| Age | 0.083 | -0.065 | 0.233 | 0.274 |
| Occupation | -0.013 | -0.083 | 0.053 | 0.722 |
| Northeast | 0.685 | 0.049 | 1.483 | 0.054 |
| Northern | -0.073 | -0.694 | 0.529 | 0.800 |
| Northwest | 0.252 | -0.903 | 1.463 | 0.652 |
| South Central | -0.002 | -0.664 | 0.578 | 0.996 |
| Southwest | -0.466 | -1.034 | 0.160 | 0.116 |

Note: 95% CI means 95% Highest posterior density (HPD) interval; L-95% CI and U-95% CI represent the upper and lower limits of 95% CI respectively; p: MCMC p-values, the probability from linear mixed models using Markov Chain Monte Carlo (MCMC) methods; *p<0.05, **p<0.01, ***p<0.001.
