# Supplementary material for: Public anxiety through various stages of COVID-19 coping: Evidence from China
Source: PLoS One. 2022 Jun 16;17(6):e0270229. doi: 10.1371/journal.pone.0270229 (PMC9202924; doi:10.1371/journal.pone.0270229)
Supplement: S10 Table — (DOCX) [file pone.0270229.s012.docx]

**S10 Table. One-way ANOVA analysis: the impact of vaccination on respondents’ risk perception, behavior, and anxiety levels**

**S10A Table. Levene’ s test for homogeneity of variance**

| Variables | Levene test | df1 | df2 | sig. |
| --- | --- | --- | --- | --- |
| Anxiety | 2.506 | 1 | 1034 | 0.114 |
| Attention domestic | 1.094 | 1 | 1034 | 0.296 |
| Controllability domestic | 32.187 | 1 | 1034 | 0 |
| Worry about being infected | 6.858 | 1 | 1034 | 0.009 |
| Interference | 1.061 | 1 | 1034 | 0.303 |
| Access to information | 2.53 | 1 | 1034 | 0.112 |
| Add worry global | 0.808 | 1 | 1034 | 0.369 |
| Attention foreign | 1.095 | 1 | 1034 | 0.296 |
| Controllability foreign | 0.754 | 1 | 1034 | 0.385 |
| Worry-Cold chain food | 0.003 | 1 | 1034 | 0.953 |
| Worry-imported goods | 0.729 | 1 | 1034 | 0.393 |
| Worry- study abroad | 2.518 | 1 | 1034 | 0.113 |
| Worry- incomes | 0.205 | 1 | 1034 | 0.651 |
| Worry- reunite | 0.35 | 1 | 1034 | 0.554 |
| Coping Behaviors | 4.388 | 1 | 1034 | 0.036 |
| Outdoor activity | 48.116 | 1 | 1034 | 0 |

**S10B Table. Results of One-way ANOVA analysis**

| Variables | sum of square | Mean Square | F | sig. |
| --- | --- | --- | --- | --- |
| Anxiety | 0.651 | 0.651 | 0.074 | 0.786 |
| Attention domestic | 1.373 | 1.373 | 1.734 | 0.188 |
| Controllability domestic | 1.638 | 1.638 | 4.199 | 0.041 |
| Worry about being infected | 0.116 | 0.116 | 0.071 | 0.791 |
| Interference | 4.762 | 4.762 | 5.989 | 0.015 |
| Access to information | 0.588 | 0.588 | 0.824 | 0.364 |
| Add worry global | 4.45 | 4.45 | 5.689 | 0.017 |
| Attention foreign | 6.939 | 6.939 | 6.626 | 0.01 |
| Controllability foreign | 3.862 | 3.862 | 3.997 | 0.046 |
| Worry-Cold chain food | 0.028 | 0.028 | 0.033 | 0.855 |
| Worry-imported goods | 0.66 | 0.66 | 0.699 | 0.403 |
| Worry- study abroad | 16.256 | 16.256 | 9.147 | 0.003 |
| Worry- incomes | 3.357 | 3.357 | 2.351 | 0.126 |
| Worry- reunite | 3.104 | 3.104 | 2.273 | 0.132 |
| Coping Behaviors | 43.228 | 43.228 | 6.849 | 0.009 |
| Outdoor activity | 7.915 | 7.915 | 20.058 | 0.000 |

**S10C Table. Mean values of perceptions, behaviors, and anxiety levels of the vaccinated/unvaccinated respondents**

| Variables | Have vaccine | Mean |
| --- | --- | --- |
| Anxiety | Yes | 6.22 |
|  | None | 6.34 |
| Attention domestic  Attention foreign | Yes | 4.19 |
|  | None  Yes  None | 4.02  3.56  3.19 |
| Controllability domestic | Yes | 4.33 |
|  | None | 4.51 |
| Worry about being infected | Yes | 3.22 |
|  | None | 3.27 |
| Interference | Yes | 3.85 |
|  | None | 3.55 |
| Access to information | Yes | 2.22 |
|  | None | 2.12 |
| Add worry global | Yes | 3.88 |
|  | None | 3.58 |
| Outdoor activity | Yes | 2.30 |
|  | None | 1.90 |
| Coping Behaviors | Yes | 5.56 |
|  | None | 6.47 |
