# Supplementary material for: Public anxiety through various stages of COVID-19 coping: Evidence from China
Source: PLoS One. 2022 Jun 16;17(6):e0270229. doi: 10.1371/journal.pone.0270229 (PMC9202924; doi:10.1371/journal.pone.0270229)
Supplement: S11 Table — (DOCX) [file pone.0270229.s013.docx]

**S11 Table. Statistics on the number of people vaccinated, vaccination willingness of respondents who hadn't been vaccinated, and the main reasons for doubts about the vaccine**

|  | N | Percent (%) |
| --- | --- | --- |
| **Have vaccine** | | |
| Yes | 54 | 5.2 |
| No | 982 | 94.8 |
| **Willingness to be vaccinated** | | |
| Yes, want to be vaccinated as soon as possible | 439 | 42.4 |
| Yes, but want to wait a while | 485 | 46.8 |
| No | 58 | 5.6 |
| **Unwillingness** | | |
| Worry about side effects | 444 | 42.9 |
| Don't believe the vaccine effect | 87 | 8.4 |
| Unnecessary, The chance of infection is extremely low | 125 | 12.1 |
| Troublesome process | 73 | 7.0 |
| Worry about being infected during the vaccination process | 112 | 10.8 |
| Healthy, the threat of the virus to yourself is limited | 44 | 4.2 |
| The COVID-19 will disappear automatically | 21 | 2.0 |
| Other | 29 | 2.8 |
