# Supplementary material for: Public anxiety through various stages of COVID-19 coping: Evidence from China
Source: PLoS One. 2022 Jun 16;17(6):e0270229. doi: 10.1371/journal.pone.0270229 (PMC9202924; doi:10.1371/journal.pone.0270229)
Supplement: S12 Table — (DOCX) [file pone.0270229.s014.docx]

**S12 Table. Socio-demographic characteristics of risk perception in each stage and throughout Stage 1 to Stage3**

**S12A Table. Socio-demographic characteristics of risk perception in the first three stages**

Note: 95% CI means 95% Highest posterior density (HPD) interval; L-95% CI and U-95% CI represent the upper and lower limits of 95% CI respectively; p: MCMC p-values, the probability from linear mixed models using Markov Chain Monte Carlo (MCMC) methods; *p<0.05, **p<0.01, ***p<0.001.

**S12B Table. Socio-demographic characteristics of risk perception from Stage 1 to Stage 3**

| Risk perceptions | Variables | Post. mean | l-95% CI | u-95% CI | p |
| --- | --- | --- | --- | --- | --- |
| Attention | Gender | 0.037 | -0.005 | 0.082 | 0.120 |
|  | Education | -0.019 | -0.038 | -0.002 | 0.054 |
|  | Age | 0.081 | 0.063 | 0.100 | 0.001 |
|  | Closing community | 0.066 | 0.002 | 0.140 | 0.044 |
|  | Occupation | 0.006 | 0.001 | 0.012 | 0.046 |
|  | Area | 0.030 | 0.011 | 0.050 | 0.001 |
| Controllability | Gender | -0.035 | -0.088 | 0.013 | 0.188 |
|  | Education | -0.051 | -0.071 | -0.031 | 0.001 |
|  | Age | 0.037 | 0.019 | 0.060 | 0.002 |
|  | Closing community | 0.107 | 0.034 | 0.195 | 0.016 |
|  | Occupation | -0.007 | -0.013 | -0.001 | 0.018 |
|  | Area | -0.003 | -0.024 | 0.021 | 0.798 |
| Knowledge | Gender | 0.030 | -0.007 | 0.067 | 0.116 |
|  | Education | 0.011 | -0.003 | 0.026 | 0.158 |
|  | Age | 0.018 | 0.002 | 0.034 | 0.024 |
|  | Closing community | 0.083 | 0.030 | 0.142 | 0.004 |
|  | Area | -0.007 | -0.012 | -0.002 | 0.002 |
|  | Occupation | 0.013 | -0.006 | 0.028 | 0.138 |
| Worried | Gender | 0.064 | 0.002 | 0.127 | 0.052 |
|  | Education | -0.160 | -0.187 | -0.134 | 0.001 |
|  | Age | 0.017 | -0.010 | 0.043 | 0.218 |
|  | Closing community | 0.094 | -0.002 | 0.193 | 0.062 |
|  | Area | 0.042 | 0.008 | 0.079 | 0.008 |
|  | Occupation | 0.015 | 0.007 | 0.023 | 0.001 |
| Trust | Gender | -0.072 | -0.125 | -0.024 | 0.004 |
|  | Education | -0.045 | -0.067 | -0.022 | 0.001 |
|  | Age | 0.043 | 0.021 | 0.064 | 0.001 |
|  | Closing community | 0.080 | -0.006 | 0.158 | 0.048 |
|  | Area | -0.014 | -0.048 | 0.016 | 0.400 |
|  | Occupation | -0.003 | -0.010 | 0.003 | 0.312 |
| Interference | Gender | -0.111 | -0.159 | -0.064 | 0.001 |
|  | Education | 0.006 | -0.015 | 0.021 | 0.530 |
|  | Age | 0.058 | 0.038 | 0.078 | 0.001 |
|  | Closing community | 0.078 | -0.005 | 0.145 | 0.042 |
|  | Area | 0.058 | 0.030 | 0.089 | 0.001 |
|  | Occupation | 0.003 | -0.003 | 0.009 | 0.348 |

Note: 95% CI means 95% Highest posterior density (HPD) interval; L-95% CI and U-95% CI represent the upper and lower limits of 95% CI respectively; p: MCMC p-values, the probability from linear mixed models using Markov Chain Monte Carlo (MCMC) methods; *p<0.05, **p<0.01, ***p<0.001.

**S12C Table. Socio-demographic characteristics of risk perceptions in Stage 4**

| Risk perceptions | Variables | Post. mean | l-95% CI | u-95% CI | p |
| --- | --- | --- | --- | --- | --- |
| Attention domestic | Gender | 0.046 | -0.054 | 0.158 | 0.386 |
|  | Education | -0.099 | -0.146 | -0.058 | 0.001 |
|  | Age | 0.093 | 0.049 | 0.131 | 0.001 |
|  | Occupation | 0.002 | -0.019 | 0.024 | 0.858 |
|  | Area | -0.024 | -0.052 | 0.007 | 0.13 |
| Attention foreign | Gender | -0.081 | -0.19 | 0.036 | 0.184 |
|  | Education | -0.15 | -0.197 | -0.106 | 0.001 |
|  | Age | 0.128 | 0.082 | 0.18 | 0.001 |
|  | Occupation | -0.003 | -0.027 | 0.018 | 0.782 |
|  | Area | -0.008 | -0.043 | 0.023 | 0.638 |
| Controllability domestic | Gender | -0.025 | -0.105 | 0.077 | 0.476 |
|  | Education | -0.006 | -0.064 | 0.033 | 0.978 |
|  | Age | 0.141 | 0.051 | 0.189 | 0.008 |
|  | Occupation | -0.029 | -0.048 | 0.005 | 0.132 |
|  | Area | 0.032 | 0.003 | 0.077 | 0.046 |
| Controllability foreign | Gender | 0.136 | 0.013 | 0.253 | 0.022 |
|  | Education | -0.087 | -0.13 | -0.035 | 0.001 |
|  | Age | 0.002 | -0.051 | 0.05 | 0.926 |
|  | Occupation | -0.004 | -0.028 | 0.019 | 0.764 |
|  | Area | 0.012 | -0.019 | 0.048 | 0.494 |
| Interference | Gender | -0.059 | -0.163 | 0.055 | 0.286 |
|  | Education | 0.007 | -0.036 | 0.047 | 0.746 |
|  | Age | -0.026 | -0.071 | 0.02 | 0.278 |
|  | Occupation | -0.012 | -0.036 | 0.007 | 0.268 |
|  | Area | 0.034 | 0.004 | 0.062 | 0.018 |
| Vaccine trust | Gender | -0.172 | -0.268 | -0.074 | 0.001 |
|  | Education | -0.121 | -0.158 | -0.083 | 0.001 |
|  | Age | 0.023 | -0.019 | 0.065 | 0.292 |
|  | Occupation | -0.008 | -0.027 | 0.011 | 0.46 |
|  | Area | -0.014 | -0.043 | 0.013 | 0.288 |
| **Worries** |  |  |  |  |  |
| Worry incomes | Gender | 0.158 | 0.021 | 0.291 | 0.02 |
|  | Education | -0.205 | -0.265 | -0.155 | 0.001 |
|  | Age | 0.024 | -0.032 | 0.087 | 0.446 |
|  | Occupation | -0.014 | -0.04 | 0.016 | 0.352 |
|  | Area | 0.035 | -0.006 | 0.071 | 0.09 |
| Worry reunite | Gender | 0.051 | -0.101 | 0.181 | 0.484 |
|  | Education | -0.046 | -0.1 | 0.017 | 0.114 |
|  | Age | -0.087 | -0.141 | -0.024 | 0.01 |
|  | Occupation | 0.017 | -0.007 | 0.047 | 0.204 |
|  | Area | 0.071 | 0.032 | 0.111 | 0.002 |
| Worry study abroad | Gender | 0.118 | -0.051 | 0.271 | 0.168 |
|  | Education | -0.115 | -0.176 | -0.053 | 0.001 |
|  | Age | 0.083 | 0.018 | 0.151 | 0.01 |
|  | Occupation | -0.005 | -0.037 | 0.027 | 0.808 |
|  | Area | 0.032 | -0.014 | 0.073 | 0.146 |
| Worry imported goods | Gender | 0.194 | 0.082 | 0.309 | 0.001 |
|  | Education | -0.075 | -0.121 | -0.033 | 0.002 |
|  | Age | 0.103 | 0.056 | 0.151 | 0.001 |
|  | Occupation | 0 | -0.022 | 0.026 | 0.996 |
|  | Area | -0.01 | -0.041 | 0.022 | 0.524 |
| Add worry global | Gender | 0.129 | 0.035 | 0.245 | 0.022 |
|  | Education | -0.109 | -0.152 | -0.06 | 0.002 |
|  | Age | 0.031 | -0.013 | 0.076 | 0.202 |
|  | Occupation | 0 | -0.021 | 0.019 | 0.98 |
|  | Area | 0.033 | 0.003 | 0.061 | 0.04 |
| Worry about being infected | Gender | 0.076 | -0.067 | 0.22 | 0.324 |
|  | Education | -0.26 | -0.317 | -0.203 | 0.001 |
|  | Age | 0.076 | 0.016 | 0.142 | 0.016 |
|  | Occupation | 0.002 | -0.028 | 0.029 | 0.874 |
|  | Area | 0.048 | 0.009 | 0.089 | 0.024 |

Note: 95% CI means 95% Highest posterior density (HPD) interval; L-95% CI and U-95% CI represent the upper and lower limits of 95% CI respectively; p: MCMC p-values, the probability from linear mixed models using Markov Chain Monte Carlo (MCMC) methods; *p<0.05, **p<0.01, ***p<0.001.
