# Supplementary material for: Public anxiety through various stages of COVID-19 coping: Evidence from China
Source: PLoS One. 2022 Jun 16;17(6):e0270229. doi: 10.1371/journal.pone.0270229 (PMC9202924; doi:10.1371/journal.pone.0270229)
Supplement: S13 Table — (DOCX) [file pone.0270229.s015.docx]

**S13 Table. Socio-demographic characteristics of coping behaviors in each stage and throughout Stage 1 to Stage3**

**S13A Table. Socio-demographic characteristics of coping behaviors in the first three stage**

Note: 95% CI means 95% Highest posterior density (HPD) interval; L-95% CI and U-95% CI represent the upper and lower limits of 95% CI respectively; p: MCMC p-values, the probability from linear mixed models using Markov Chain Monte Carlo (MCMC) methods; *p<0.05, **p<0.01, ***p<0.001.

**S13B Table. Socio-demographic characteristics of coping behaviors Stage 1 to Stage 3**

| Coping behaviors | Variables | Post. mean | l-95% CI | u-95% CI | p |
| --- | --- | --- | --- | --- | --- |
| Precaution | Gender | 0.048 | 0.009 | 0.086 | 0.028 |
|  | Education | -0.019 | -0.035 | -0.002 | 0.022 |
|  | Age | -0.010 | -0.025 | 0.007 | 0.282 |
|  | Closing community | 0.153 | 0.102 | 0.221 | 0.001 |
|  | Area | 0.000 | -0.023 | 0.024 | 0.934 |
|  | Occupation | 0.003 | -0.001 | 0.009 | 0.188 |
| Protective behavior | Age | 0.091 | 0.040 | 0.145 | 0.001 |
|  | Gender | 0.454 | 0.326 | 0.571 | 0.001 |
|  | Education | -0.131 | -0.182 | -0.083 | 0.001 |
|  | Occupation | 0.001 | -0.014 | 0.017 | 0.924 |
|  | Closing community | 0.174 | -0.012 | 0.366 | 0.064 |
|  | Area | 0.119 | 0.062 | 0.189 | 0.001 |
| Outdoor activity | Age | 0.118 | 0.060 | 0.169 | 0.001 |
|  | Gender | -0.576 | -0.703 | -0.442 | 0.001 |
|  | Education | -0.109 | -0.162 | -0.057 | 0.001 |
|  | Occupation | 0.031 | 0.013 | 0.047 | 0.001 |
|  | Closing community | -0.498 | -0.692 | -0.275 | 0.001 |
|  | Area | 0.181 | 0.046 | 0.319 | 0.010 |
| Access to information | Age | 0.082 | 0.060 | 0.105 | 0.001 |
|  | Gender | -0.040 | -0.096 | 0.013 | 0.150 |
|  | Education | 0.032 | 0.012 | 0.054 | 0.004 |
|  | Occupation | 0.001 | -0.005 | 0.008 | 0.748 |
|  | Closing community | 0.131 | 0.052 | 0.216 | 0.002 |
|  | Area | 0.012 | -0.012 | 0.038 | 0.380 |

Note: 95% CI means 95% Highest posterior density (HPD) interval; L-95% CI and U-95% CI represent the upper and lower limits of 95% CI respectively; p: MCMC p-values, the probability from linear mixed models using Markov Chain Monte Carlo (MCMC) methods; *p<0.05, **p<0.01, ***p<0.001.

**S13C Table. Socio-demographic characteristics of coping behaviors in Stage 4**

| Coping behaviors | Variables | Post. mean | l-95% CI | u-95% CI | p |
| --- | --- | --- | --- | --- | --- |
| Protective behavior | Gender | 0.636 | 0.357 | 0.923 | 0.001 |
|  | Education | -0.357 | -0.467 | -0.232 | 0.001 |
|  | Age | 0.246 | 0.134 | 0.369 | 0.001 |
|  | Occupation | -0.002 | -0.055 | 0.055 | 0.970 |
|  | Area | 0.035 | -0.051 | 0.107 | 0.398 |
| Outdoor activity | Gender | -0.832 | -1.200 | -0.454 | 0.001 |
|  | Education | -0.020 | -0.169 | 0.110 | 0.790 |
|  | Age | -0.236 | -0.393 | -0.061 | 0.008 |
|  | Occupation | 0.159 | 0.083 | 0.229 | 0.001 |
|  | Area | 0.119 | 0.014 | 0.234 | 0.040 |
| Access to information | Gender | -0.039 | -0.145 | 0.061 | 0.436 |
|  | Education | -0.112 | -0.148 | -0.075 | 0.001 |
|  | Age | 0.109 | 0.067 | 0.148 | 0.001 |
|  | Occupation | 0.003 | -0.016 | 0.022 | 0.812 |
|  | Area | 0.001 | -0.028 | 0.027 | 0.916 |

Note: 95% CI means 95% Highest posterior density (HPD) interval; L-95% CI and U-95% CI represent the upper and lower limits of 95% CI respectively; p: MCMC p-values, the probability from linear mixed models using Markov Chain Monte Carlo (MCMC) methods; *p<0.05, **p<0.01, ***p<0.001.
