# Supplementary material for: Public anxiety through various stages of COVID-19 coping: Evidence from China
Source: PLoS One. 2022 Jun 16;17(6):e0270229. doi: 10.1371/journal.pone.0270229 (PMC9202924; doi:10.1371/journal.pone.0270229)
Supplement: S1 Text — (DOCX) [file pone.0270229.s016.docx]

**S1 Text. Considering both fixed effects and random effects GLMM**

Taking account of the random effects caused by continuous tracking and to capture the robust predictors, we uniquely created an overall GLMM for the first three adjacent stages to verify the results of the GLMM constructed separately in each stage. The model was implemented by R package MCMCglmm, which was created based on Monte Carlo Markov Chain (MCMC) algorithm [1]. GLMM is able to deal with the multicollinearity in models and avoid over fitting and can effectively adapt to the non-normally data as well as consider both fixed and random effects at the same time. Variables like individual factors (age, gender, area, etc.), risk perception and coping behaviors were classified as fixed effects. The variable ID was treated as random effect to control because of repeated sampling [2].

**Reference**

1 Hadfield, J. D. MCMC methods for multi-response generalized linear mixed models: the MCMCglmm R package. Journal of statistical software 33, 1-22, doi:10.18637/jss.v033.i02 (2010).

2 Dingemanse, N. J. & Dochtermann, N. A. Quantifying individual variation in behaviour: mixed-effect modelling approaches. Journal of Animal Ecology 82, 39-54, doi:10.1111/1365-2656.12013 (2013).
