# Supplementary material for: Public anxiety through various stages of COVID-19 coping: Evidence from China
Source: PLoS One. 2022 Jun 16;17(6):e0270229. doi: 10.1371/journal.pone.0270229 (PMC9202924; doi:10.1371/journal.pone.0270229)
Supplement: S2 Text — (DOCX) [file pone.0270229.s017.docx]

**S2 Text. Descriptive statistics of all participants**

5,983 valid questionnaires (effective sampling: 87.3%) were used for analysis. The numbers of valid questionnaires for the four stages were 2,087, 1,731, 1,129 and 1,036, respectively. The test of internal consistency reliability and structural validity in the four-stage questionnaire were good (Cronbach's α > 0.7, KMO > 0.7, Bartlett’s test: p < 0.05, see S2 Table for specific test results). Respondents came from Mainland China and were divided into six regions. Male and female respondents accounted for 43.0% and 57.0%, respectively. 26.9% of respondents didn’t have a bachelor’s degree. Respondents aged 18-25 accounted for 51.6%. More detailed demographic information is shown in S3 Table.
