# Supplementary material for: Public anxiety through various stages of COVID-19 coping: Evidence from China
Source: PLoS One. 2022 Jun 16;17(6):e0270229. doi: 10.1371/journal.pone.0270229 (PMC9202924; doi:10.1371/journal.pone.0270229)
Supplement: S3 Text — (DOCX) [file pone.0270229.s018.docx]

**S3 Text. Socio-demographic characteristics of risk perception and coping behaviors**

We further examined the Socio-demographic characteristics of risk perception in each stage as well as the joint influences in the first three stages (Fig 3, S12 Table). People who were older or had lower education levels tended to have a high overall risk perception regarding COVID-19 (S12 Table). These people also were more concerned about the pandemic and trusted more in the government’s prevention and control measures. In each stage of the pandemic, the elderly always paid more attention to COVID-19 related information especially during the peak stage (Post. mean = 0.25, 95% CI 0.19 to 0.31, p < 0.001) while the less educated people were more worried about being infected (p < 0.001). Female respondents showed significantly higher levels of attention to COVID-19 than males at the initial outbreak (Post. mean = 0.21, 95% CI 0.10 to 0.35, p < 0.01). The female was also more worried about being infected (Post. mean = 0.12, 95% CI 0.01 to 0.21, p < 0.05) and had less confidence in domestic control of COVID-19 (Post. mean = -0.09, 95% CI -0.16 to -0.02, p < 0.01) than the male at the peak of the pandemic. In Stage 4, among different risk perceptions concerning domestic COVID-19, gender difference was only significant in the trust of vaccines with females displaying much lower levels of trust (Post. mean = -0.17, 95% CI -0.27 to -0.07, p < 0.001). Compared with those with higher education levels, the less educated also expressed more worries (Post. mean = -0.26, 95% CI -0.32 to -0.20, p < 0.001) and paid more attention to the new waves of outbreak (Post. mean = -0. 10, 95% CI -0.15 to -0.06, p < 0.001) in Stage 4. In the same stage, the lower educated also showed lower trust in vaccines in Stage 4 (Post. mean = -0.17, 95% CI -0.27 to -0.07, p < 0.001).
